# CDK contribution to DSB formation and recombination in fission yeast meiosis

**DOI:** 10.1371/journal.pgen.1007876

**Published:** 2019-01-14

**Authors:** Luisa F. Bustamante-Jaramillo, Celia Ramos, Leticia Alonso, Aroa Sesmero, Mónica Segurado, Cristina Martín-Castellanos

**Affiliations:** 1 Instituto de Biología Funcional y Genómica, Consejo Superior de Investigaciones Científicas, Salamanca, Spain; 2 Instituto de Biología Funcional y Genómica and Departamento de Microbiología y Genética, Universidad de Salamanca, Salamanca, Spain; Comenius University in Bratislava, SLOVAKIA

## Abstract

CDKs (cyclin-dependent kinases) associate with different cyclins to form different CDK-complexes that are fundamental for an ordered cell cycle progression, and the coordination of this progression with different aspects of the cellular physiology. During meiosis programmed DNA double-strand breaks (DSBs) initiate recombination that in addition to generating genetic variability are essential for the reductional chromosome segregation during the first meiotic division, and therefore for genome stability and viability of the gametes. However, how meiotic progression and DSB formation are coordinated, and the role CDKs have in the process, is not well understood. We have used single and double cyclin deletion mutants, and chemical inhibition of global CDK activity using the *cdc2-asM17* allele, to address the requirement of CDK activity for DSB formation and recombination in fission yeast. We report that several cyclins (Cig1, Cig2, and the meiosis-specific Crs1) control DSB formation and recombination, with a major contribution of Crs1. Moreover, complementation analysis indicates specificity at least for this cyclin, suggesting that different CDK complexes might act in different pathways to promote recombination. Down-regulation of CDK activity impinges on the formation of linear elements (LinEs, protein complexes required for break formation at most DSB hotspot sites). This defect correlates with a reduction in the capability of one structural component (Rec25) to bind chromatin, suggesting a molecular mechanism by which CDK controls break formation. However, reduction in DSB formation in cyclin deletion mutants does not always correspondingly correlate with a proportional reduction in meiotic recombination (crossovers), suggesting that specific CDK complexes might also control downstream events balancing repair pathways. Therefore, our work points to CDK regulation of DSB formation as a key conserved feature in the initiation of meiotic recombination, in addition to provide a view of possible roles CDK might have in other steps of the recombination process.

## Author summary

Meiotic division is a cell division process where a single round of DNA replication is followed by two sequential chromosome segregations, the first reductional (homologous chromosomes separate) and the second equational (sister chromatids segregate). As a consequence diploid organisms halve ploidy, producing haploid gametes that after fertilization generate a new diploid organism with a complete chromosome complement. At early stages of meiosis physical exchange between homologous chromosomes ensures the accurate following reductional segregation. Physical exchange is provided by recombination that initiates with highly-controlled self-inflicted DNA damage (DSBs, double strand breaks). We have found that the conserved CDK (cyclin-dependent kinase) activity controls DSB formation in fission yeast. Available data were uncertain about the conservation of CDK in the process, and thus our work points to a broad evolutionary conservation of this regulation. Regulation is exerted at least by controlling chromatin-binding of one structural component of linear elements, a protein complex related to the synaptonemal complex and required for high levels of DSBs. Correspondingly, depletion of CDK activity impairs formation of these structures. In addition, CDK might control homeostatic mechanisms, critical to maintain efficient levels of recombination across the genome and, therefore, high rates of genetic exchange between parental chromosomes.

## Introduction

Eukaryotic cell cycle progression is driven by sequentially organized accumulation of different CDK (cyclin-dependent kinase) activities formed by a catalytic serine/threonine kinase that binds to a regulatory cyclin subunit [1–3]. In *Schizosaccharomyces pombe* a single CDK (Cdc2) and six different cyclins have been described. Cig1, Cig2 and Puc1 cyclins control G1 progression, meanwhile Cdc13 is essential to promote chromosome segregations [4–9]. Though at least in this yeast a single CDK complex (Cdc2 kinase-Cdc13 cyclin) can promote both mitotic and meiotic progression, it is not as efficient as in the wild-type situation where additional CDK complexes are present [8, 9]. This indicates that distinct cyclins have evolved to optimize different aspects of the mitotic and the meiotic divisions and that some kind of specificity is provided by each CDK complex (Cdc2-Cyclin). Indeed, two of the described cyclins (Rem1 and Crs1) are meiosis-specific [10, 11], suggesting meiosis-specific functions for these CDK-complexes.

Meiosis is a special cell division where a single round of DNA replication is followed by two rounds of chromosome segregation. In the first reductional segregation homologous chromosomes separate apart, and the physical links provided by recombination between the pair of homologs is required for the orientation in the meiotic spindle, and therefore for their successful segregation [12]. Thus, a key feature of meiosis is self-inflicted DNA double-strand break (DSB) formation that initiates natural recombination at specific genome locations known as hotspots [13]. Meiotic DSBs are generated by a conserved topoisomerase II-like protein, Spo11 (Rec12 in fission yeast), assisted by a group of accessory proteins forming the conserved pre-recombination complexes SFT and DSBC in fission yeast [13, 14]. In addition to the DSB machinery, DSB formation requires a meiosis-specific chromosome context, provided by histone variant H2A.Z, meiosis-specific cohesin subunits (Rec8 and Rec11 in fission yeast), and Linear Elements (structurally related to the axial/lateral elements of the synaptonemal complex of other eukaryotes) [15–18]. Indeed, meiotic cohesins are required for LinE formation and chromosome loading of LinE components [14–16, 19–21]; specifically, casein kinase 1-dependent phosphorylation of the meiotic cohesin subunit Rec11 is required for the interaction with the LinE-component Rec10 and LinE formation [22, 23].

After break formation Spo11 (Rec12) covalently linked to DNA is endonucleolytically removed and resection generates single-stranded DNA (ssDNA) tracts that, coated with strand-exchange proteins Rad51/Dmc1, invades homologous chromosome for homology search and repair [24–30]. This invasion generates by strand displacement and DNA synthesis the so-called D-loop that can be dissolved, and the invading nucleoprotein filament annealed with its sister chromatid in the original chromosome (synthesis-dependent strand annealing), resulting in non-reciprocal exchange between the parental chromosomes (non-crossovers, NCOs). Alternatively, the D-loop can be stabilized and mature into recombination intermediates, Holliday junctions. The way these intermediates are resolved by structure-dependent endonucleases will generate crossovers (COs, reciprocal exchange between the pair of homologs) or NCOs [31–33]. Thus, the fate of the D-loop is an important point for CO/NCO regulation. Stabilization of the invading nucleoprotein filament promotes D-loop stabilization and therefore CO formation, meanwhile the counteractions of helicases dissolve the D-loop and therefore hamper CO formation [33–36]. CO homeostasis and CO invariance mechanisms have been proposed to maintain minimal levels of COs and their regular distribution along chromosomes [37–40].

DSB formation occurs after meiotic DNA replication during meiotic prophase. In budding yeast when DNA replication is locally delayed, DSB formation is also locally retarded, pointing to the coordination between both events [41]. Moreover, in fission yeast local changes in origin selection during meiotic DNA replication lead to local changes in the distribution of meiotic recombination [42]. It was proposed that replication-origin firing leads to the recruitment of recombination factors. This hypothesis was recently supported in budding yeast. In this yeast, kinase activities involved in cell cycle regulation are important for DSB formation. S-phase specific CDK and DDK (Dbf4-dependent kinase) activities phosphorylate Mer2 (fission yeast Rec15 ortholog, component of the conserved SFT-complex). Mer2 phosphorylation promotes the interaction with other Spo11-accessory proteins, and is essential for association of Spo11 with hotspots and DSB formation [43–46]. A link between Mer2 phosphorylation and DNA replication has not formally been established; however, replication-fork passage correlates with chromosome loading of Rec114 (a Spo11-accessory protein whose chromatin association depends on Mer2 phosphorylation) (fission yeast Rec7 ortholog) that may promote the local formation of the pre-recombination complexes [30, 47–49].

However, DNA replication *per se* is not necessary for DSB formation since inhibition of S-phase initiation by down-regulation of replication factors, both in budding and fission yeast, does not abrogate DSB formation [50–52]. Thus, this coordination depends on an active DNA replication and, indeed, the S-phase checkpoint blocks DSB formation when replication is stalled [53, 54]. In fission yeast checkpoint inhibition of DSB formation works, at least in part, by repressing the expression of the transciption factor gene *mei4* which, in turn, controls the expression of *mde2* (coding for one of the Rec12-accessory proteins) [14, 55, 56]. In budding yeast, among other mechanisms, checkpoint activation down-regulates DDK activity, thereby preventing Mer2 phosphorylation and DSB formation [54]. Unscheduled DSB formation on partially replicated chromosomes generates unrepaired breaks that hamper further replication, and impinges on cell viability [54].

DSB formation is a key conserved feature of meiosis and, apart from the sequence conservation of Spo11 homologs, proteins of the pre-recombination complexes have amino acid similarity and some of them are even structurally conserved among different species [14, 57–60], suggesting that regulation by cell cycle kinases could be also a conserved feature. In fission yeast, DDK activity is also required for DSB formation and recombination but the nature of this regulation is currently unknown [61, 62]. In the case of CDK, DSB formation is still observed when CDK activity is down-regulated [50]; however, DSBs were not quantified in that study and CDK requirement was not addressed. We have studied the role of CDK activity in DSB formation in fission yeast by analyzing the effects of the depletion of CDK complexes normally present in meiotic prophase. We have found that Cig1, Cig2, and the meiosis-specific Crs1 cyclin control indeed DSB formation and recombination, with a major contribution of Crs1, and the stronger reduction of DSBs in the double deletion mutant *cig1 crs1*. At least for Crs1, complementation analysis of the recombination phenotype by increasing copy number of other cyclins and Cdc2 suggests specificity. The absence of these cyclins reduces binding to chromatin of the LinE-component Rec25, and impairs the maturation of these structures. We have obtained similar results when global CDK activity was down-regulated using an ATP-analog sensitive *cdc2-asM17* allele. This study points to CDK regulation of DSB formation as a conserved feature in the initiation of meiotic recombination. Furthermore, comparison of DSB, NCO and CO levels suggests that CDK activity might also control downstream events after DSB formation. Therefore, CDK activity in meiosis may regulate different steps of the recombination process.

## Results

### Cyclin deletion mutants reduce meiotic recombination

Meiotic DSB formation was previously analyzed in fission yeast using a temperature- sensitive *cdc2* mutant, *cdc2-L7* [50]. Using a thermal induction of meiosis in haploid cells, DNA breaks were visualized by separating chromosomes in pulsed-field gel electrophoresis (PFGE) and evaluating chromosome fragmentation during meiotic prophase. Rec12-dependent chromosome breakage was observed after meiotic induction at the restrictive temperature, indicating that Cdc2 is not essential to initiate meiotic recombination. However, since DSBs were not quantified in the study, the contribution of CDK to DSB formation was not evaluated. We have revisited this result and, since Cdc2 is essential for meiotic progression, decided to analyze first the recombination efficiency of cyclin deletion mutants. We have focused on cyclins that, with a clear temporal expression pattern (mRNA, protein, and/or associated kinase activity) during meiosis, may play a role in recombination. Cig1, Cig2 and Crs1 cyclins were selected based on this criterion. Cig1 and Cig2 cyclins are expressed around S-phase and prophase, and Cig2 required for meiotic DNA replication (http://www.pombase.org/spombe/result/SPCC4E9.02 and http://www.pombase.org/spombe/result/SPAPB2B4.03; expression viewer and pombeTV) [63, 64]. The cyclin-related protein Crs1 shows a very high level of expression during meiotic prophase and was identified in our initial screening for mutants affecting meiotic chromosome segregation, compatible with a recombination defect (http://www.pombase.org/spombe/result/SPBC2G2.09c; http://telecic.cicancer.org/pombe/) [65]. In the absence of Cig1, Cig2, and Puc1 cyclins, Crs1 contributes to meiotic G1 progression [9]. Meanwhile Cig1 and Cig2 are also expressed and contribute to mitotic progression [4, 5], Crs1 shows a complex regulation to prevent RNA accumulation in vegetative cells [66, 67]; indeed, mis-expression of this cyclin in mitotic cycles causes segregation problems and lethality [10, 68]. Though expressed later in meiosis and involved in meiosis I entry, the other meiosis-specific cyclin Rem1 has an additional role in meiotic recombination. However, this does not depend on the presence of the cyclin-box in the protein, suggesting that it is Cdc2-independent [11, 69]; therefore, we did not include it in this study. In the case of *puc1*, expression is almost undetectable during meiotic prophase, where it is even less abundant than in vegetative cells (https://www.pombase.org/gene/SPBC19F5.01c).

**Fig 1:**
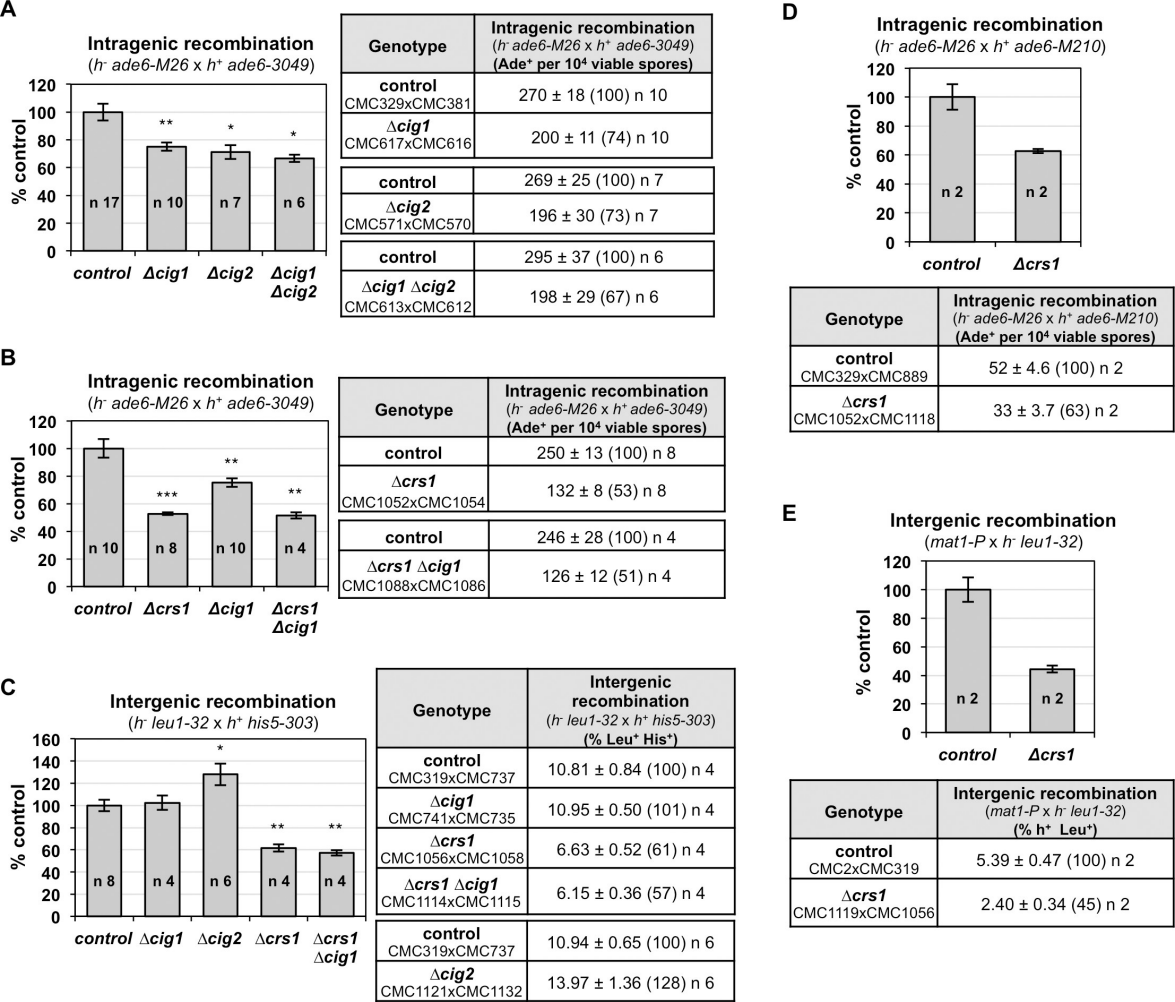
Cyclin deletion mutants impair meiotic recombination. **(A) and (B)** Crosses of *h*^*-*^ *ade6-M26* x *h*^*+*^ *ade6-3049* were performed in MEA and plated for recombinant frequency at least twice. Table on the right shows gene conversion expressed as the mean of Ade^+^ per 10^4^ viable spores +/- SEM of n independent crosses based on the cumulative number of spore colonies in each cross; 91–1236 Ade^+^ colonies scored in each independent cross. The numbers in parentheses are percentages relative to wild-type control. Strains used in the crosses are indicated. Graph on the left shows gene conversion expressed as mean of the percentage relative to the control cross +/- SEM of n independent crosses. Each mutant was analyzed only with its control cross in the same experiment. **(C)** Crosses of *h*^*-*^ *leu1-32* x *h*^*+*^ *his5-303* in MEA were performed and plated for recombinant frequency twice. Table on the right shows crossover levels expressed as the mean of the percentage of recombinants +/- SEM of n independent crosses based on the cumulative number of spore colonies in each cross; 667–2763 haploid spore colonies analyzed in each plating. The numbers in parentheses are percentages relative to wild-type control. Strains used in the crosses are indicated. Graph on the left shows crossovers expressed as mean of the percentage relative to the control cross +/- SEM of n independent crosses. Each mutant was analyzed only with its control cross in the same experiment. **(D)** Crosses of *h*^*-*^ *ade6-M26 Δcrs1* x *h*^*+*^ *ade6-M210 Δcrs1* in MEA were performed and plated for recombinant frequency twice. Data representation as in (A) of 2 independent crosses based on the cumulative number of spore colonies in each cross; 580–1131 Ade^+^ colonies scored in each independent cross. **(E)** Crosses of *h*^*+*^ *Δcrs1* x *h*^*-*^ *leu1-32 Δcrs1* in MEA were performed and plated for recombinant frequency twice. Data representation as in (C) of 2 independent crosses based on the cumulative number of spore colonies in each cross; more than 965 haploid spore colonies analyzed in each plating. *p* values were calculated based on Student´s t-test (unpaired, two tails), * <0.05, ** ≤0.01, *** ≤0.001.

Meiotic recombination was addressed both in intragenic (NCOs measured as gene conversion at *ade6* on chromosome III) and intergenic (COs in the *leu1-his5* interval on chromosome II) recombination assays in single and double deletion mutants. Single deletion mutants of *cig1* and *cig2* were defective in recombination, with a moderate reduction in gene conversion (26% *p* value 0.004 and 27% *p* value 0.016, respectively), and without additive effects in the double *cig1 cig2* mutant (33% reduction; *p* value 0.025), suggesting that both cyclins might act in the same genetic pathway (Fig 1A). Regarding COs (Fig 1C), *cig1* deletion mutants did not show a defect and levels of crossovers were similar to the control. Interestingly, levels of crossovers in the *cig2* deletion mutant were even higher than in the control cross (1.28-fold higher; *p* value 0.034). In the case of the meiosis-specific Crs1 cyclin, *crs1* deletion mutants showed stronger defects both in gene conversion, with 53% of the gene conversion level shown in the control (*p* value 2.1 10^−6^), and in COs, with 61% of the control levels (*p* value 0.004) (Fig 1B and 1C). Finally, we analyzed the double *cig1 crs1* mutant, and found that recombination levels were similar to those in the single *crs1* mutant, with 51% of the gene conversion and 56% of the CO level shown in the control (*p* value 0.006 and 0.002, respectively) (Fig 1B and 1C), indicating the strong phenotype of the *crs1* deletion. This reduction in recombination does not have an impact on spore viability (S1 Fig). Crs1 was recently reported not to have a role in meiotic recombination using different markers for the recombination assays [9]. Since mutants defective in chromosome architecture, such as meiosis-specific cohesin mutants and LinE-mutants, have a regional defect in recombination [15–17, 70, 71], we decided to address recombination in the genetic intervals used in that study. The deletion of *crs1* reduced recombination, both gene conversion and crossovers, at *ade6* (*ade6-M26 ade6-M210* interval) and *mat1-leu1* interval, to a similar extent as in our previous recombination assays with a 63% of the gene conversion and 45% of the crossover levels shown in the control crosses (Fig 1D and 1E). These genetic data indicate that Cig1, Cig2, and Crs1 cyclins control meiotic recombination.

### CDK activity contributes to meiotic DSB formation

**Fig 2:**
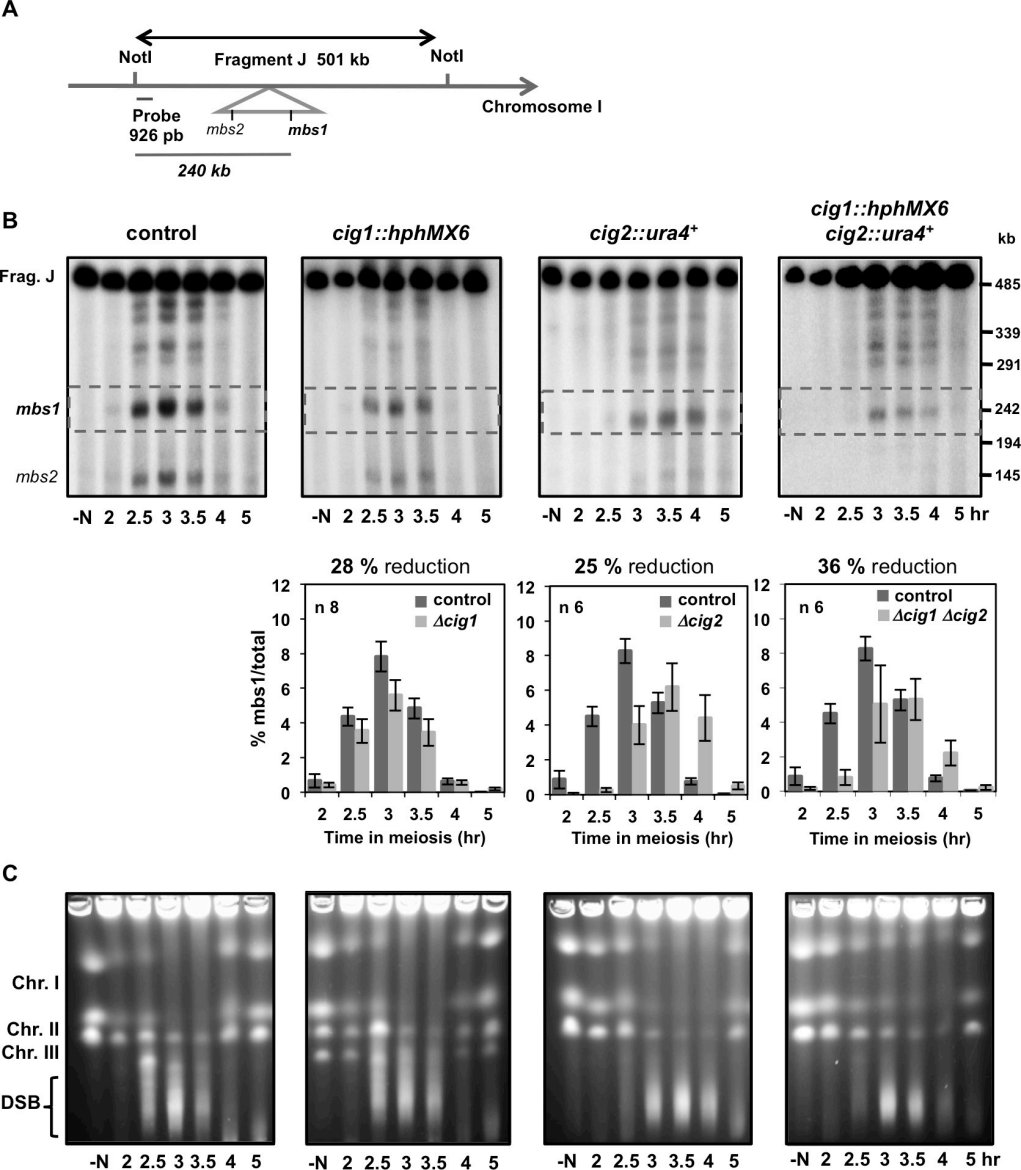
DSB formation in *cig1*, *cig2*, and *cig1 cig2* deletion mutants. **(A)** Scheme of chromosome position of the *mbs1* hotspot and probe used for break detection. **(B)** Detection of *mbs1* breakage by Southern blot in control (CMC7), *cig1* (CMC1010), *cig2* (CMC1022), and double *cig1 cig2* (CMC1023) deletion mutants during synchronous meiosis of *pat1-114* diploids; representative blots are shown. Below each blot the percentage of breakage is represented as the mean +/- SEM of n independent experiments. Each mutant was analyzed with the control kinetics in the same experiment. *p* values (maximal percentage of breakage) were calculated based on Student´s t-test (unpaired, two tails): 0.094 *cig1* mutant, 0.210 *cig2* mutant, and 0.060 double *cig1 cig2* mutant. **(C)** PFGE separation of entire chromosomes during meiotic kinetics. DSBs are visualized as a transient smear below the chromosomes. Control and *cig1* experiments were analyzed in the same gel and therefore similarly stained and subjected to the same image processing. *cig2* and double *cig1 cig2* mutants were analyzed in the same gel and therefore similarly stained and subjected to the same image processing. Images were adjusted to show similar signal in chromosomes at the initial time point (-N).

The recombination defect led us to analyze DSB formation at the strong natural hotspot *mbs1* on chromosome I (Fig 2A) [72]. Diploid cyclin-deletion mutants, previously G1-arrested by nitrogen depletion, were induced to enter meiosis synchronously by thermal induction using a temperature-sensitive allele of the meiotic inhibitor Pat1 (*pat1-114)* [73]. DSB formation was analyzed by Southern blot with DNA samples from cells collected at different time points before and after the induction, including time points when DSBs were expected to form and be repaired (Fig 2B). In the control experiment DSBs at *mbs1* were detected from 2 to 4 hr after meiotic induction and then started to disappear due to break repair. Clear levels of breakage were observed from 2.5–3.5 hr, corresponding to cells in prophase according to flow cytometry and nuclear counting (S2 Fig), with a maximum of 7.8% breakage at 3 hr (Fig 2B).

**Fig 3:**
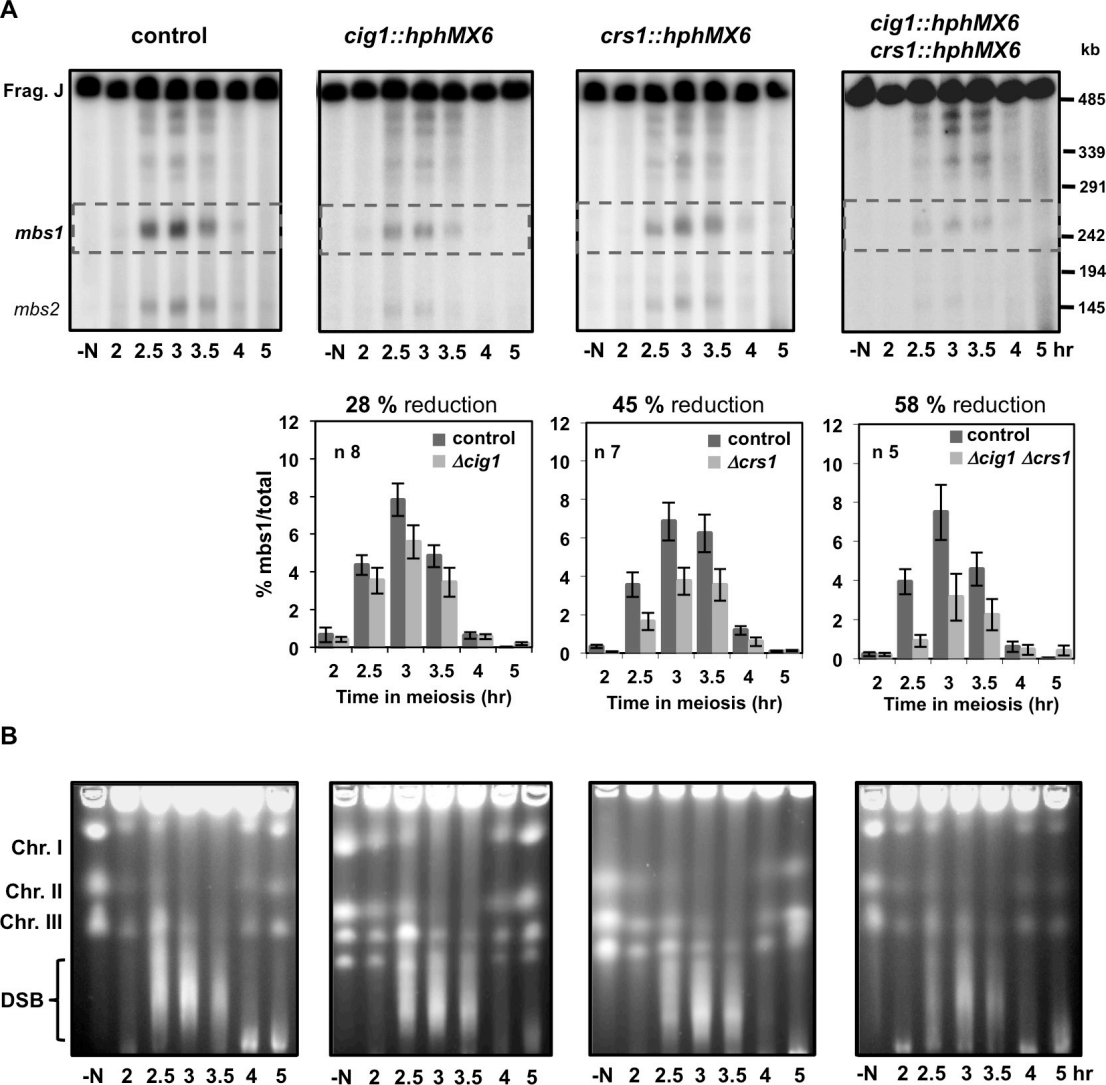
DSB formation in *cig1*, *crs1*, and *cig1 crs1* deletion mutants. **(A)** Detection of *mbs1* breakage by Southern blot in control (CMC7), *cig1* (CMC1010), *crs1* (CMC1059), and double *cig1 crs1* (CMC1113) deletion mutants during synchronous meiosis of *pat1-114* diploids; representative blots are shown. Below each blot the percentage of breakage is represented as the mean +/- SEM of n independent experiments. Each mutant was analyzed with the control kinetics in the same experiment. *p* values (maximal percentage of breakage) were calculated based on Student´s t-test (unpaired, two tails): 0.094 *cig1* mutant, 0.025 *crs1* mutant, and 0.048 double *cig1 crs1* mutant. *p* value for *crs1* mutant: 0.033 at 2.5 hr, 0.025 at 3 hr, and 0.058 at 3.5 hr. *p* value for double *cig1 crs1* mutant: 0.003 at 2.5 hr, 0.048 at 3 hr, and 0.080 at 3.5 hr. **(B)** PFGE separation of entire chromosomes during meiotic kinetics. DSBs are visualized as a transient smear below the chromosomes. Control and *cig1 crs1* mutant were analyzed in the same gel and therefore similarly stained and subjected to the same image processing. Images were adjusted to show similar signal in chromosomes at the initial time point (-N).

The kinetics of DSB appearance and disappearance at *mbs1* in *cig1* deleted cells was similar to that in the control; however, though not statistically significant, levels of breakage were reduced by 28% compared to the control levels (*p* value 0.094), with a maximum of 5.6% breakage at 3 hr. Similar results were obtained in *cig2* deletion mutants with a maximum of breakage of 6.2% (25% reduction, 8.3% maximum control levels; *p* value 0.210), in this case at 3.5 hr since this mutant showed a delay in S-phase entry and progression (S2 Fig). The double mutant *cig1 cig2* showed a reduction in DSB formation similar to that in the single deletion mutants, with a breakage of 5.3% (36% reduction, 8.3% maximum control levels, *p* value 0.060) (1–0.72x0.75 = 0.46 expected reduction for an additive effect). Reduction in DSB formation was also analyzed genome-wide by entire chromosome visualization. DSB formation appears during prophase as a smear below the intact chromosomes that disappears after repair at later time points. Although this type of analysis is not quantitative, *cig1*, *cig2*, and *cig1 cig2* deletion mutants seemed to reduce the smear intensity suggesting a possible general reduction in DSB formation in these mutants (Fig 2C). DSB levels were similarly analyzed in *crs1* deletion mutants (Fig 3 and S3 Fig). *crs1* mutants progressed through meiosis with the same timing as the wild-type strain (S4 Fig) and DSB formation was clearly detected from 2.5–3.5 hr after meiotic induction (Fig 3A); however, levels of breakage were statistically reduced by 45% of the level in the control experiment (*p* value 0.025), with a maximum of 3.7% breakage at 3 hr (6.9% maximum control levels). The double mutant *cig1 crs1* showed a stronger reduction in DSB formation and breaks at *mbs1* were reduced 58%, with a maximum level of 3.1% at 3 hr (7.5% maximum control levels, *p* value 0.048) (1–0.72x0.55 = 0.60 expected reduction for an additive effect). The defect in DSB formation of *crs1* and the double *cig1 crs1* mutant were also visible when entire chromosomes were analyzed (S3 Fig and Fig 3B). This physical analysis of the cyclin deletion mutants indicates that Crs1, and probably Cig1 and Cig2, control DSB formation.

**Fig 4:**
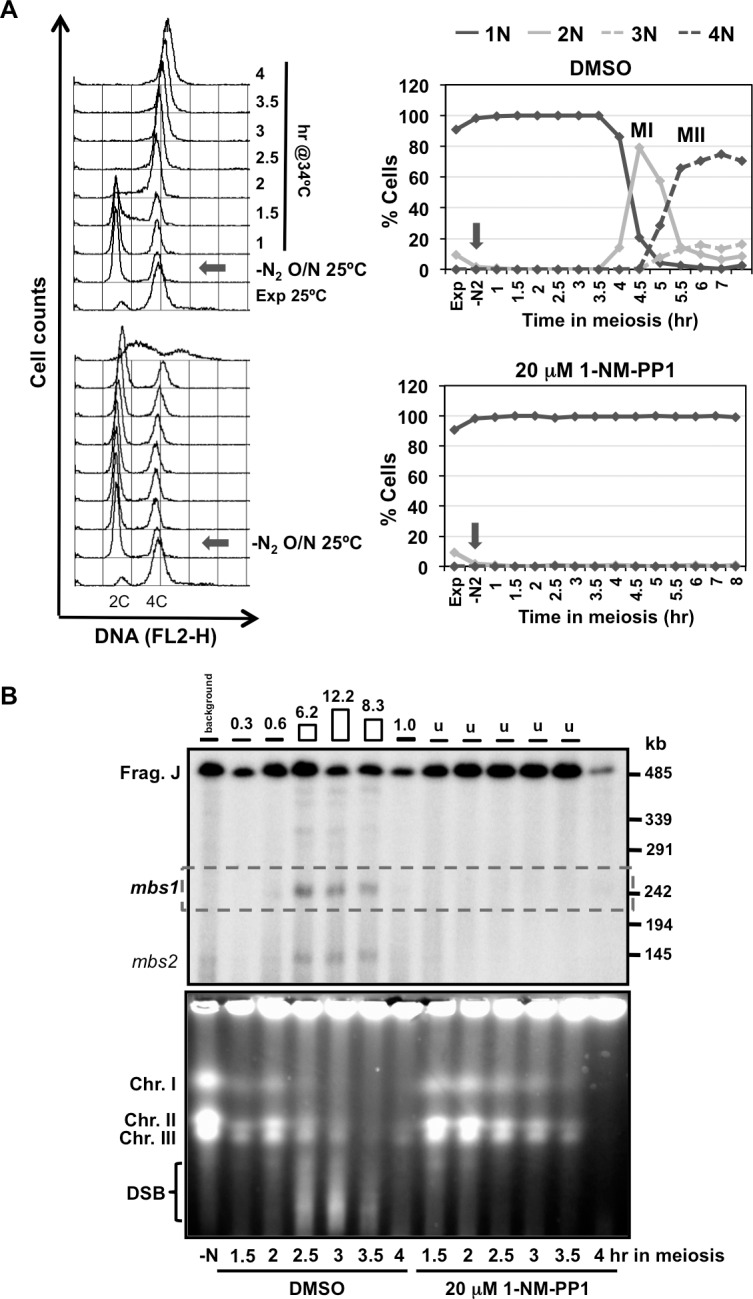
Chemical inhibition of Cdc2 blocks DSB formation (CDK inhibition at meiotic entry). **(A) On the left** Flow cytometry analysis of synchronous diploid *pat1-114 cdc2-asM17* meiosis (CMC1066) with DMSO (top panel) or 20 μM 1-NM-PP1 (bottom panel) added at the beginning of meiotic induction (arrows). DNA content (FL2-H) histograms are shown. **On the right** Quantification of chromosome segregation by DAPI staining and nuclear counting (1 nucleus, 2 nuclei, 3 nuclei, and 4 nuclei) is shown. Timing of meiosis I (MI) and meiosis II (MII) is indicated. Arrows indicate time of DMSO or 1-NM-PP1 addition. Partial inactivation of the ATP-analog at later time points (4 hr) allows DNA replication (compare DNA profiles on the left graphs for DMSO and 1-NM-PP1 treated cells) without chromosome segregation. **(B) Top panel** Detection of *mbs1* breakage by Southern blot during the same meiotic kinetics. Percentage of breakage in the control DMSO experiment is indicated on top; breakage after 1-NM-PP1 addition was undetectable (<0.1%). Similar result (inhibition of DSB formation at *mbs1* hotspot) was obtained in an independent experiment. **Bottom panel** PFGE separation of entire chromosomes during the same meiotic kinetics. DSBs are visualized as a transient smear below the chromosomes. Similar result (genome-wide inhibition of DSB formation) was obtained in an independent experiment.

**Fig 5:**
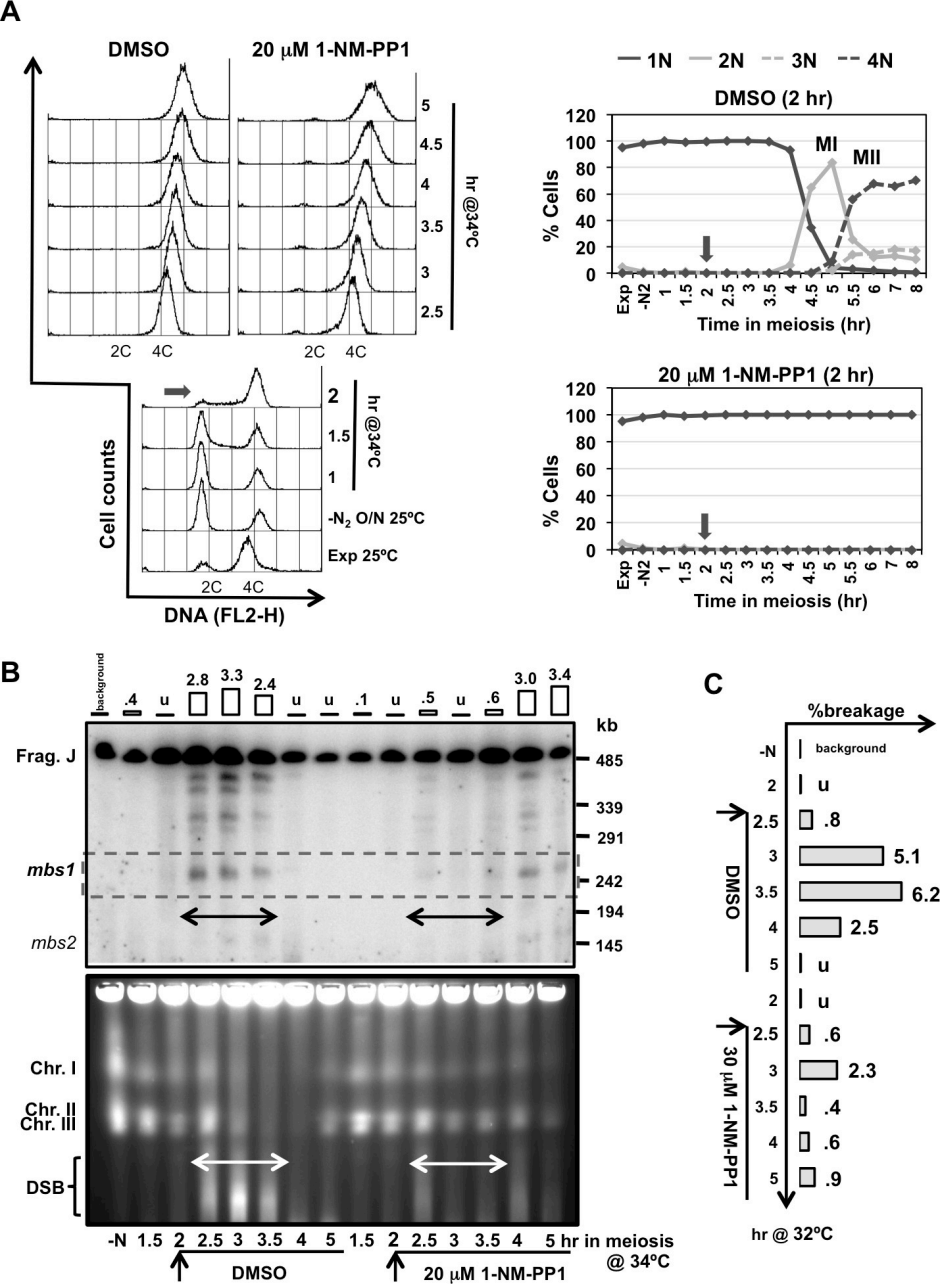
Chemical inhibition of Cdc2 blocks DSB formation (CDK inhibition after DNA replication). **(A) On the left** Flow cytometry analysis of synchronous diploid *pat1-114 cdc2-asM17* meiosis (CMC1066) with DMSO (top left panel) or 20 μM 1-NM-PP1 (top right panel) added at 2 hr (arrow in the bottom panel) after meiotic induction of a common culture. DNA content (FL2-H) histograms are shown. **On the right** Quantification of chromosome segregation by DAPI staining and nuclear counting (1 nucleus, 2 nuclei, 3 nuclei, and 4 nuclei) is shown. Timing of meiosis I (MI) and meiosis II (MII) is indicated. Arrows indicate time of DMSO or 1-NM-PP1 addition. **(B) Top panel** Detection of *mbs1* breakage by Southern blot during the same meiotic kinetics. Double-headed arrows indicate natural temporal position (2.5–3.5 hr) of DSB formation. Percentage of breakage is indicated on top; u undetectable (<0.1%). Partial inactivation of the ATP-analog at later time points (4 hr) allows DSB formation without chromosome segregation. Similar result (significant inhibition of DSB formation at *mbs1* hotspot) was obtained in an independent experiment at 32°C with 30 μM 1-NM-PP1 added at 2.5 hr after meiotic induction (quantification shown in **C**). **(B) Bottom panel** PFGE separation of entire chromosomes during the same meiotic kinetics. DSBs are visualized as a transient smear below the chromosomes. Double-headed arrows indicate natural temporal position (2.5–3.5 hr) of DSB formation. Similar result (genome-wide significant inhibition of DSB formation) was obtained in an independent experiment.

Next, we decided to revisit the requirement of Cdc2 for DSB formation using an inhibition of global CDK activity by means of the ATP-analog sensitive *cdc2-asM17* allele [74]. When 1-NM-PP1 ATP-analog was added at the beginning of the time course in cells arrested in G1, just before thermal induction of entry into meiosis, S-phase entry and chromosome segregation were blocked as expected for the inhibition of Cdc2-kinase activity (Fig 4A); and more importantly, DSB formation was undetectable at *mbs1* and the other break sites in the NotI J fragment (Fig 4B, top panel). The impact on DSB formation after the inhibition of Cdc2 activity was also observed when entire chromosomes were analyzed. No smears below the intact chromosomes were detected compared to the control experiment (Fig 4B, bottom panel). A similar result was obtained when the ATP-analog was added later, at 2 hr after meiotic induction when cells were exiting S-phase and progressing into prophase: DSB formation was significantly reduced and chromosome segregation was blocked (Fig 5). This was particularly clear at the initial time points of the experiment (indicated by a double-headed arrow in Fig 5B) when DSBs were normally detected in the control. DSB formation was activated at later time points probably due to analog inactivation (see also legend of Fig 4A). Since replication defects trigger the S-phase checkpoint to block DSB formation [53, 56], we performed the experiment using the *rad3* deletion mutant (coding for the apical sensor kinase in the checkpoint signaling pathway) [75] to exclude the possibility that the absence of DSB formation results from checkpoint activation due to incomplete replication upon Cdc2 inhibition. Again, a significant reduction in DSB formation at hotspots in the NotI J fragment was observed after inhibition of Cdc2 even when checkpoint activation was abrogated (S5 Fig). These results complement the previous results and support a role of CDK activity in meiotic DSB formation in fission yeast.

### CDK activity is required for maturation of LinEs and efficient chromatin association of Rec25

**Fig 6:**
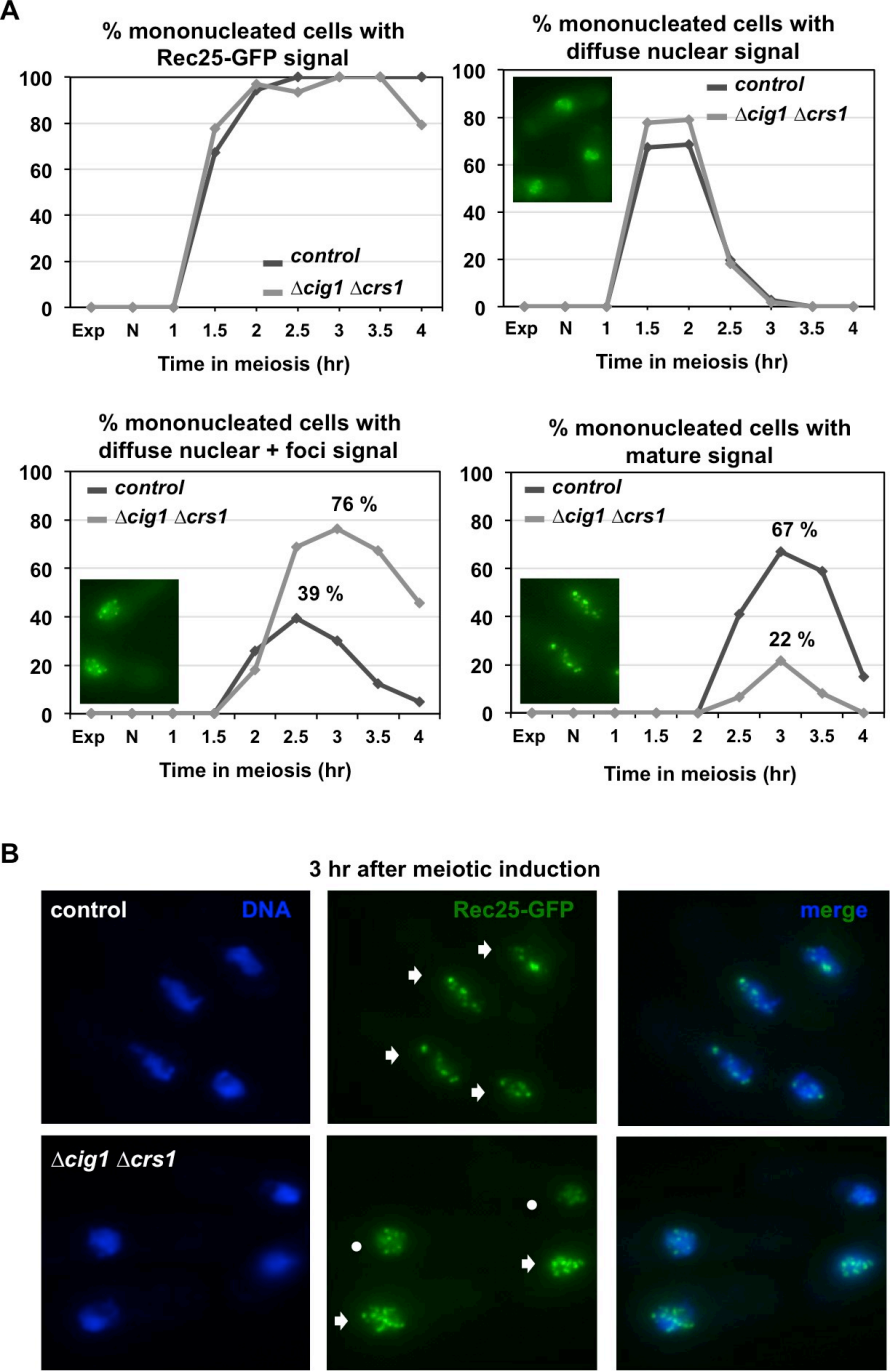
Defective LinE maturation in double *cig1 crs1* deletion mutants. **(A)** Quantification of LinE formation by visualization of Rec25-GFP maturation status during synchronous diploid *pat1-114 rec25-GFP* meiosis of control (CMC78) and double *cig1 crs1* deletion mutants (CMC1207). Graphs represent the percentage of mono-nucleated cells with Rec25-GFP signal (any category), diffuse nuclear signal (earliest transient category visible at S-phase), diffuse nuclear+foci signal (later transient category visible at prophase), and mature signal (clear foci; latest category visible at late prophase). An example of each category is shown in the inset picture in the corresponding graph. **(B)** Photographs of cells in prophase (Methanol/Acetone fixed) at 3 hr after meiotic induction when maximal maturation of LinEs is observed in the control. DNA (DAPI-staining; left panels), Rec25-GFP signal (middle panels), and both (merge; right panels). Circles indicate cells with Rec25-GFP diffuse nuclear+foci signal and arrows cells with mature signal. At least 45 cells were analyzed at each time point from 1.5 hr after meiotic induction (when signal first appeared), increasing the number of cells to 49–73 during prophase.

DSB formation at nearly all hotspots requires LinEs. When these structures are absent, in deletion mutants of their components, DSB formation at the NotI J fragment containing the hotspot *mbs1* is extremely impaired and, in the cases genome-wide analyzed, DSB formation at most hotspots is abolished [15, 17, 65, 76]. Therefore, we addressed LinE formation as a possible point of regulation by CDK activity. LinE formation was visualized in intact cells during synchronous diploid *pat1-114* meiosis using a Rec25-GFP version, and was first analyzed in double *cig1 crs1* deletion mutants that as shown above exhibit the stronger reduction in DSB formation (Fig 6). Synchrony of the experiment was followed by cytometry and counting of nuclei, and as described above (S4 Fig) double *cig1 crs1* cells showed a normal timing of meiotic events (S-phase, MI and MII entry) (S6A Fig). Rec25-GFP signal was quantified by counting the percentage of cells with no signal, diffuse nuclear signal, diffuse nuclear signal plus foci, and mature signal (clear foci with no background diffuse signal), following the natural dynamics of LinE formation in intact cells (see Fig 6A and S6B Fig for cells representing each category) [15]. The kinetics of accumulation of Rec25-GFP signal was similar in control and *cig1 crs1* cells, reaching almost 100% of the population during prophase (Fig 6A). Similarly, the earliest transient signal (diffuse nuclear signal) appeared and disappeared with the same kinetics during S-phase (1.5–2.5 hr; see S6A Fig for meiotic progression). However, although the next transient signal (diffuse nuclear+foci) appeared at the same time in *cig1 crs1* mutant and control cells, it accumulated in a higher proportion (76% of the cells compared to the 39% in the control experiment), and disappeared later. Moreover, the latest signal (mature signal) reached 67% of the population at 3 hr after meiotic induction in the control experiment, but in the *cig1 crs1* deletion mutant was observed in only 22% of the cells (Fig 6A and 6B, S6B Fig). This result indicates that the maturation of LinEs is defective in the absence of Cig1 and Crs1 cyclins.

**Fig 7:**
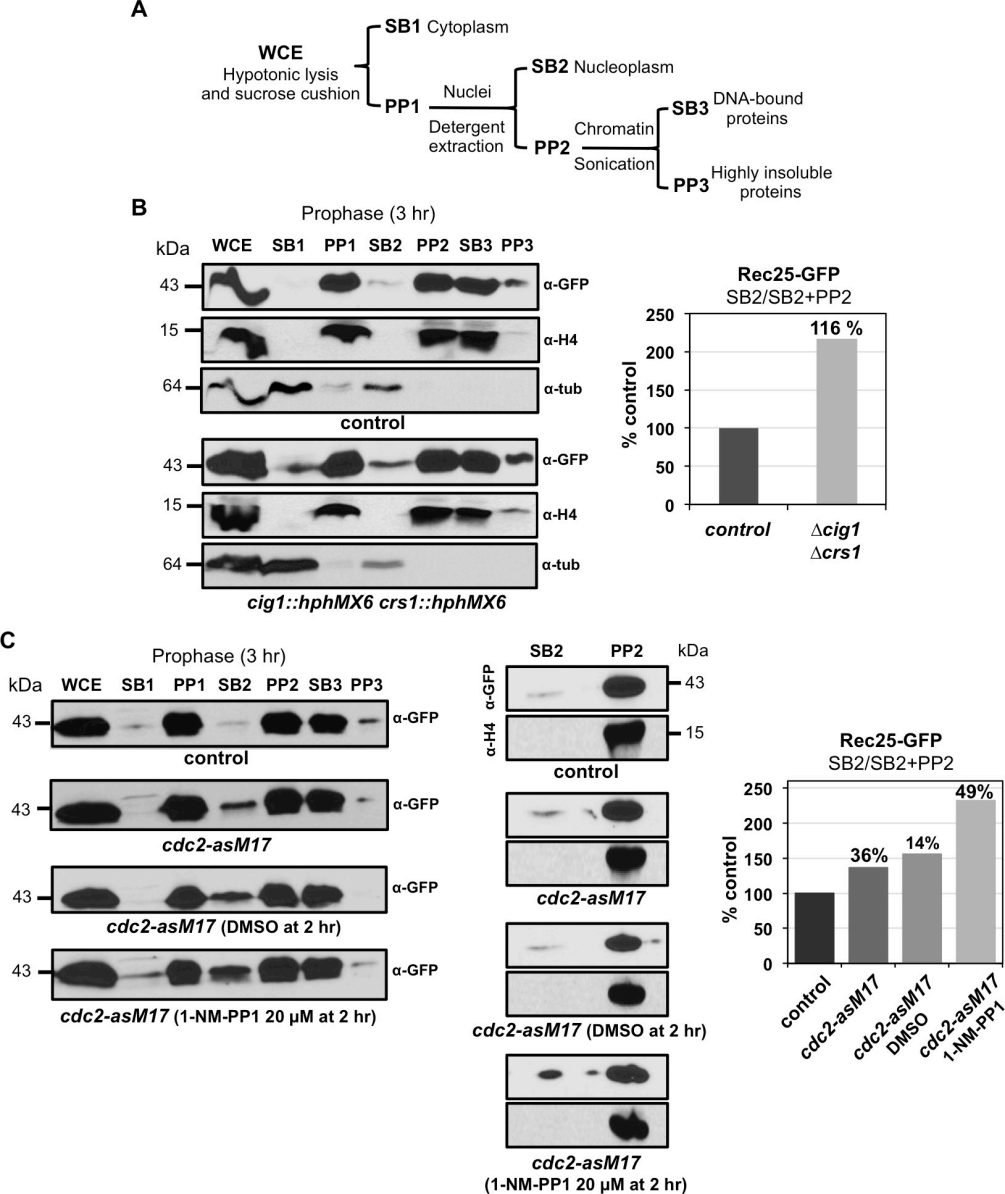
CDK down-regulation reduces chromatin binding of the LinE-component Rec25. Synchronous diploid *pat1-114 rec25-GFP* meiosis were induced and cells collected in prophase for cellular fractionation following the scheme shown in **(A)**. **(B)** Rec25-GFP chromatin binding is reduced in double *cig1 crs1* deletion mutants. Synchronous diploid *pat1-114 rec25-GFP* meiosis of control (CMC78) and double *cig1 crs1* deletion mutants (CMC1207) were induced and cells collected after 3 hr for cellular fractionation. **On the left** Western blot analysis of the different fractions. The distribution of Rec25-GFP protein was followed with anti-GFP antibodies. The same membranes were used to analyze the distribution of the Histone H4 and Tubulin as controls. Each lane contains equal extract equivalents (7% of WCE). **On the right** Quantification of Rec25-GFP recovery after detergent treatment of the nuclear fraction (SB2/SB2+PP2), expressed as the percentage relative to the control strain. For quantification only SB2 and PP2 fractions were loaded in a new gel leaving an empty lane in between to avoid sample mixing by diffusion. Similar result (increased Rec25-GFP solubilization, 1.66 fold) was obtained in an independent experiment. Quantification in WCE of Rec25-GFP normalized to histone H4 indicates similar Rec25-GFP levels in control and *cig1 crs1* mutant cells (ratio control/mutant 0.96 in the experiment shown, and 1.13 in the second independent experiment). **(C)** Chemical inhibition of Cdc2 reduces Rec25-GFP chromatin binding (CDK inhibition after DNA replication). Synchronous diploid *pat1-114 rec25-GFP cdc2-asM17* meiosis (CMC1192) were induced, DMSO or 20 μM 1-NM-PP1 added at the end of S-phase (at 2 hr), and cells collected for cellular fractionation 1 hr later (at 3 hr after meiotic induction). As additional controls, untreated *cdc2-asM17* cells and a *cdc2*^*+*^ strain (CMC78) were also analyzed. **On the left** Western blot analysis as in B of the different fractions. **On the right** Quantification of Rec25-GFP recovery after detergent treatment of the nuclear fraction (SB2/SB2+PP2). As in B only SB2 and PP2 fractions were loaded in a new gel avoiding sample mixing by diffusion (western blots on the left). The same membranes were used to analyze the distribution of the Histone H4 as a control. Graph on the right shows solubilization as the percentage relative to the control strain. Numbers on the columns indicate the increment relative to their respective controls: wild-type strain for *cdc2-asM17*, *cdc2-asM17* for *cdc2-asM17* DMSO treated, and *cdc2-asM17* DMSO treated for *cdc2-asM17* 1-NM-PP1 treated. Similar result (increased Rec25-GFP solubilization after ATP-analog treatment, 1.46-fold) was obtained in an independent experiment.

We noticed that at late time points in prophase, 3.5 and 4 hr after meiotic induction, cells with a tangled Rec25-GFP signal were frequently present in the *cig1 crs1* mutant (27% and 38% respectively compared to 4% and 5% in the control cells) (S6B Fig). The defect in LinE maturation and the frequency of this tangled signal prompted us to perform a cellular fractionation assay to address the ability of Rec25-GFP to bind chromatin in the absence of Cig1 and Crs1 (Fig 7). Briefly, cells were collected in prophase at 3 hr after meiotic induction when LinEs reach their maximal maturation stage, treated with zymolyase for cell wall digestion, and subjected to a hypotonic lysis. The whole extracts (WCE) were centrifuged through a sucrose cushion to collect the cytoplasmic (SB1) and the nuclear (PP1) fractions. Nuclear fractions were treated with detergent to solubilize the nucleoplasmic proteins, and centrifuged to separate into soluble fractions (SB2) and the nuclear-insoluble fractions (PP2) that were gently sonicated for further solubilization generating the final SB3 and PP3 fractions, corresponding to chromatin-bound proteins and highly insoluble nuclear proteins (Fig 7A). In the control experiment, Rec25-GFP protein was detected in the same fractions as the Histone H4 (PP1, PP2, and SB3), indicating its capability to bind chromatin and its resistance to detergent extraction (Fig 7B, left). However, although Histone H4 was detected in the same fractions in the double *cig1 crs1* deletion mutant, the amount of Rec25-GFP substantially increased in the nucleoplasmic fraction (SB2), 2.16-fold compared to the control (Fig 7B, right). Thus, in the absence of Cig1 and Crs1 cyclins the binding to chromatin of the LinE-component Rec25 is less efficient.

**Fig 8:**
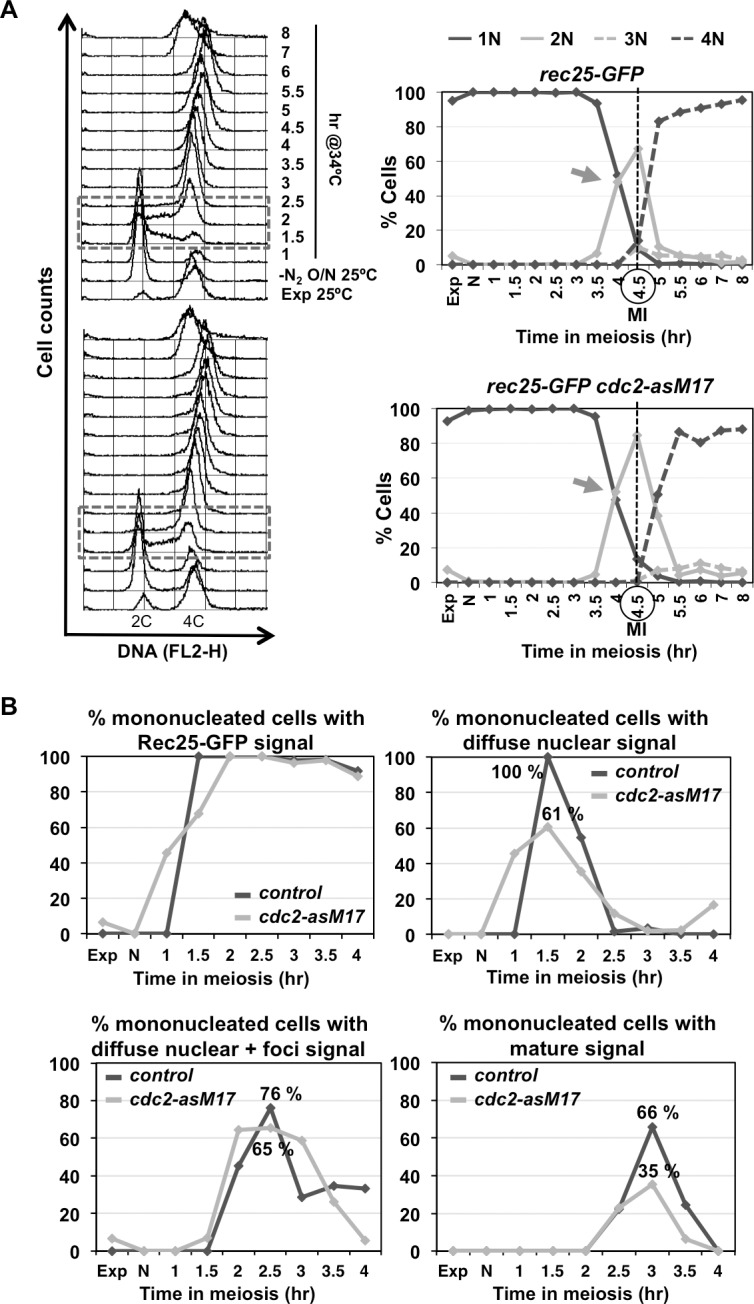
Defective LinE maturation in *cdc2-asM17* mutants without treatment. Synchronous diploid *pat1-114 rec25-GFP* meiosis of control (CMC78) and *cdc2-asM17* mutant (CMC1192) were induced. **(A)** Meiotic progression. **On the left** Flow cytometry analysis showing DNA content (FL2-H) histograms. Dashed-lined box outlines premeiotic S-phase progression. **On the right** Quantification of chromosome segregation by DAPI staining and nuclear counting (1 nucleus, 2 nuclei, 3 nuclei, and 4 nuclei) is shown. The arrows indicate meiosis I (MI) entry, and the vertical dashed-lines indicate the peak of MI. **(B)** Quantification of LinE formation by visualization of Rec25-GFP maturation status during the same meiotic kinetics. Graphs represent the percentage of mono-nucleated cells with Rec25-GFP signal (any category), diffuse nuclear signal (earliest transient category visible at S-phase), diffuse nuclear + foci signal (later transient category visible at prophase), and mature signal (clear foci; latest category visible at late prophase). At least 28 cells were analyzed at each time point from 1 hr after meiotic induction (when signal first appeared), increasing the number of cells to 46–91 during prophase.

**Fig 9:**
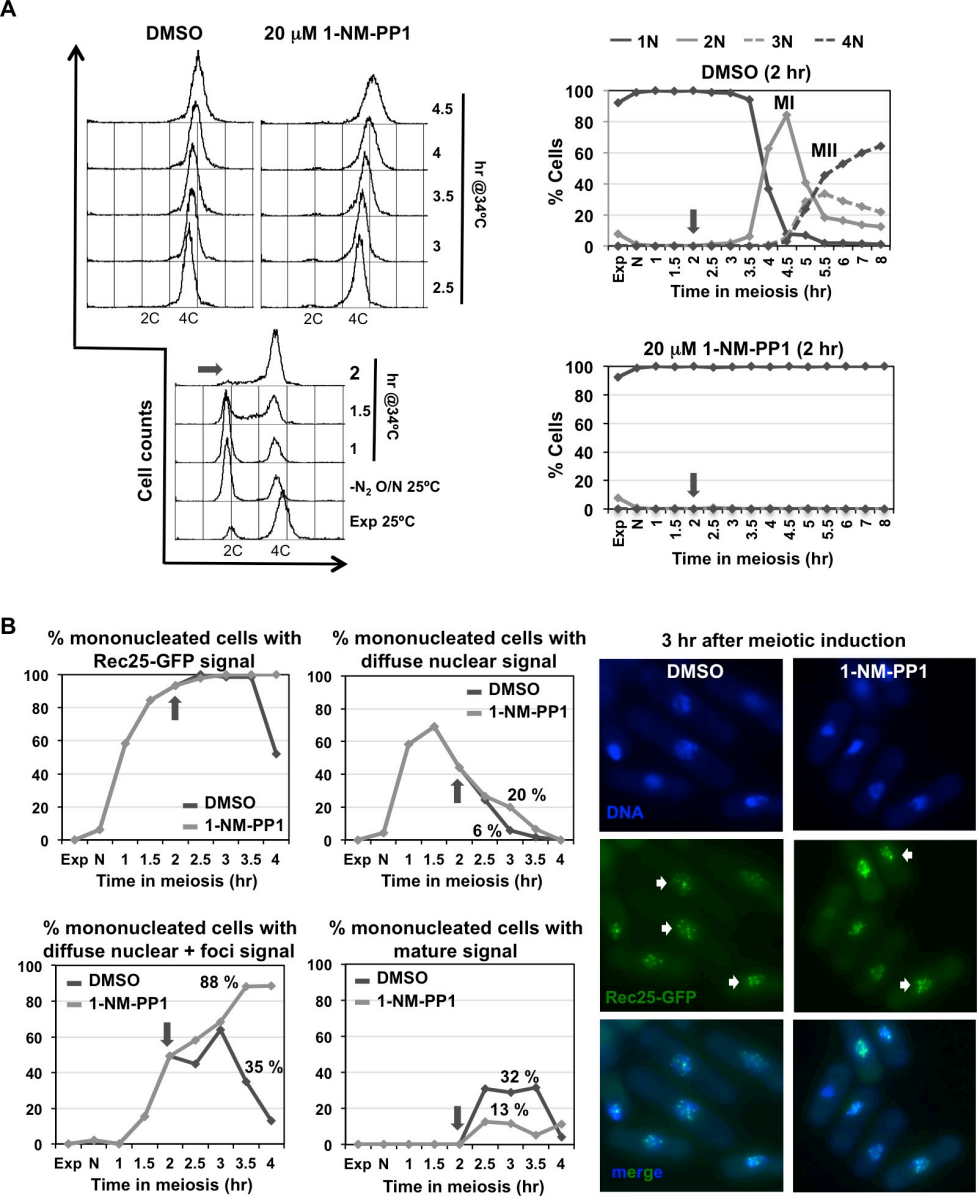
Chemical inhibition of Cdc2 impairs LinE maturation (CDK inhibition after DNA replication). **(A) On the left** Flow cytometry analysis of synchronous diploid *pat1-114 cdc2-asM17* meiosis (CMC1192) with DMSO (top left panel) or 20 μM 1-NM-PP1 (top right panel) added at 2 hr (arrow in the bottom panel) after meiotic induction of a common culture. DNA content (FL2-H) histograms are shown. **On the right** Quantification of chromosome segregation by DAPI staining and nuclear counting (1 nucleus, 2 nuclei, 3 nuclei, and 4 nuclei) is shown. Timing of meiosis I (MI) and meiosis II (MII) is indicated. Arrows indicate time of DMSO or 1-NM-PP1 addition. **(B) On the left** Quantification of LinE formation by visualization of Rec25-GFP maturation status during the same meiotic kinetics. Graphs represent the percentage of mono-nucleated cells with Rec25-GFP signal (any category), diffuse nuclear signal (earliest transient category visible at S-phase), diffuse nuclear + foci signal (later transient category visible at prophase), and mature signal (clear foci; latest category visible at late prophase). Arrows indicate time (2 hr) of DMSO or ATP-analog addition. **Right panel** Photographs of cells in prophase (Methanol/Acetone fixed) at 3 hr after meiotic induction when maximal maturation of LinEs is observed in the control. DNA (DAPI-staining; top panels), Rec25-GFP signal (middle panels), and both (merge; bottom panels). Arrows indicate cells with Rec25-GFP mature signal; notice the heterogeneity of Rec25-GFP categories in 1-NM-PP1 treated cells. At least 36 cells were analyzed at each time point from 1 hr after meiotic induction (when signal first appeared), increasing the number of cells to 60–93 during prophase. Similar result (defective LinE maturation) was obtained in an independent experiment.

Both LinE maturation and Rec25-GFP chromatin binding were similarly analyzed using the *cdc2-asM17* allele to control global CDK inactivation in prophase. Interestingly, maturation defects and reduced chromatin binding were also observed in the *cdc2-asM17* mutant without treatment (Figs 7C and 8), and these defects were more pronounced when CDK activity was depleted by the ATP-analog (Figs 7C and 9). In the experiment without treatment, the kinetics of appearance and disappearance of the earliest transient signal (diffuse nuclear signal) was sharper in the wild-type control. Rec25-GFP signal was observed earlier in the *cdc2-asM17* mutant than in the control (Fig 8B), probably due to the faster S-phase progression of the mutant and advanced Rec25-GFP expression (S7 Fig and Fig 8A). At 1 hr after meiotic induction 46% of the population already exhibited this signal, whereas in the control it did not appear until 1.5 hr; however, only 61% of the population in the *cdc2-asM17* mutant showed the diffuse nuclear signal at 1.5 hr after meiotic induction compared to 100% in the wild-type control. Despite this fact, the kinetics of appearance and disappearance of the following transient signal (diffuse nuclear+foci) was similar in control and *cdc2-asM17* mutant cells, as well as meiosis I entry (Fig 8A and 8B), indicating an extended prophase in the mutant. Both control and mutant cells showed a similar proportion of cells with this signal at 2.5 hr after meiotic induction (76% and 65% respectively). However, at 3 hr only 35% of the mutant population exhibited the latest mature signal compared to the 66% of the wild-type control. This suggests that expression and nuclear localization of Rec25-GFP are normal in the *cdc2-asM17* mutant in which lower CDK activity has been reported [74], but maturation of LinEs into clear nuclear foci is compromised.

Next, we studied LinE formation after inhibition of global CDK activity by addition of 1-NM-PP1 when cells were exiting S-phase and progressing into prophase (2 hr after meiotic induction; same experimental design as the one described above for the study of DSB formation) (Fig 9A). ATP-analog addition caused a reproducible delay (in two independent experiments) in the disappearance of the transient diffuse nuclear signal; at 3 hr after meiotic induction 20% of the treated population showed this type of signal compared to 6% of the DMSO-treated control cells (Fig 9B). The next transient signal (diffuse nuclear+foci) reached a similar proportion in both situations at 3 hr; however, in 1-NM-PP1 treated cells the signal did not progress properly and, instead of decreasing at later times points as observed in the control population, continued to accumulate. This population showed a high proportion (76% at 3.5 hr) of cells with tangled Rec25-GFP signal compared to the DMSO treated control (7%). More importantly, Rec25-GFP mature signal was observed in only 13% of the cells compared to 32% in the control.

Regarding chromatin-binding capability of Rec25-GFP protein, we observed an increase of 1.36-fold in the nucleoplasmic fraction (SB2) in the *cdc2-asM17* untreated mutant compared to the wild-type control, and an increase of 1.49-fold in the *cdc2-asM17* 1-NM-PP1 treated mutant compared with the DMSO treated control (Fig 7C). Similar results, in terms of LinE maturation and Rec25-GFP susceptibility to detergent treatment, were obtained in an independent experiment. Thus, the results using the *cdc2-asM17* mutant are consistent with the ones obtained in the double *cig1 crs1* deletion mutant, and support a role for CDK activity in the formation/maturation of LinEs.

### Possible CDK targets to control DSB formation

In an effort to identify CDK substrates involved in DSB formation we have done direct mutagenesis of several proteins essential for break formation at hotspots: Rec10 (LinEs), Rec27 (LinEs), Rec7 (SFT-complex) and Rec14 (DSBC-complex) [14, 15, 17, 65, 77] (S8 Fig). Given the implication of CDK activity in LinE maturation reported here, we paid special attention to LinE components when generating mutants. Of the four LinE components described only the phosphoprotein Rec10 [78] and Rec27 harbor CDK phosphorylation sites, 8 and 1 respectively. We thought phosphoprotein Rec7 (SFT-complex) [14] could be also a good candidate since the S245 in the TSSPFN context is adjacent to T243 and S244, which are potential sites for DDK activity, resembling the S28-S29-S30 cluster of amino acids in Mer2 that are subjected to CDK-priming phosphorylation, and subsequent DDK phosphorylation [44, 45]. Finally, we selected the conserved Rec14 protein (DSBC-complex) harboring 5 CDK sites. We have changed the putative CDK phosphorylated residues (in minimal S/T-P and consensus S/T-P-X-K/R/(N) context) to alanine generating phospho-null mutants at these residues of these proteins. These residues show a good prediction score in PhosphoNet 2.0 and/or Phospho Yeast 1.0 software, and in some cases (S347, T482, and S529 residues in Rec10) are phosphorylated *in vivo* during meiotic prophase [78]. The mutants were genetically analyzed to score for defects in gene conversion in intragenic recombination assays. None of the mutants impaired recombination rates, except for *rec14 (cdk1)* mutants where a moderate 20% reduction was observed (S9 Fig). Recombination rates were not further reduced in the *rec14 (cdk total)* mutant harboring mutations in all the putative CDK phosphorylated residues. Furthermore, combinations of some of these mutants (*rec7*, *rec14*, *and rec27*) did not reduce gene conversion in qualitative recombination assays (S10 Fig).

## Discussion

It is not well established that control of DSB formation by CDK activity is a universal feature of meiosis. Addressing this issue in the fission yeast *S*. *pombe*, we have found that *cig1* and *cig2* cyclin deletion mutants are indeed impaired in meiotic recombination, and NCOs reduced 26% compared to the control levels observed in wild-type strains. Correspondingly, DSB formation is also reduced to a similar extent at the hotspot of reference *mbs1*, 28% and 25% respectively. Non-additive defects in NCO and DSB levels were observed in the double *cig1 cig2* mutant, indicating these CDK-complexes may act in the same genetic pathway (Fig 1A and Fig 2). However, CO formation was not correspondingly diminished, and in the case of *cig2* mutants CO levels were even increased (Fig 1C). This result suggests that the moderate reduction in DSBs may be real and that in the absence of these CDK-complexes the reduced DSB levels activate homeostatic mechanisms to maintain CO levels, since they are essential to ensure a successful segregation of chromosomes and, therefore, for the viability of the meiotic products. This phenomenon, known as crossover homeostasis, has been described in several organisms, including budding and fission yeast [37, 38, 40]. Alternatively, given the increase of COs in *cig2* mutants, these CDK-complexes may control downstream events in the recombination process. One possibility is that they negatively control CO formation by regulating the stability of the D-loop after homolog invasion. In fission yeast it has been proposed that Rad51/Dmc1 accessory proteins protect the D-loop from the unwinding action of helicases, promoting in this way the formation of Holliday junctions and COs [33, 34, 36]. In this view, Cdc2-Cig2 (and Cdc2-Cig1 to a minor extent) could phosphorylate and inhibit accessory proteins required for nucleoprotein filament stabilization and strand-exchange activity [79–82], impairing D-loop stability. Different phosphoproteomic approaches have identified S/T phosphorylated residues in the Rad51/Dmc1-accesory protein Sfr1 in vegetative cells [83–86]; four of them are putative CDK phosphorylation sites, and at least one of them (S165) is phosphorylated in a Cdc2-dependent manner during mitotic M-phase [86]. Alternatively, Cdc2-Cig2 (and Cdc2-Cig1 to a minor extent) could phosphorylate and activate Fml1 (or other helicases counteracting D-loop formation) [33, 36]. As Sfr1, Fml1 harbors putative phosphorylation sites by Cdc2; however, in this case phosphorylation of these residues has not been reported in phosphoproteomic studies.

In addition to Cig1 and Cig2, the meiosis-specific Crs1 cyclin is also required for DSB formation and recombination, in this case to a greater extent. In the absence of Crs1 both NCOs and COs are similarly reduced in the different tested intervals, 37–47% and 39–55% respectively (Fig 1B, 1C, 1D and 1E). This reduction correlates well with a corresponding 45% reduction in DSB formation both in proficient and deficient DSB-repair conditions (Fig 3 and S3 Fig). The proportional reduction of NCOs and COs indicates that the levels of DSBs observed in this mutant could be under the threshold level to activate CO homeostasis. Interestingly, DSBs in double *cig1 crs1* deletion mutants are further reduced (58%). Although not statistically significant compared to the DSB levels in the single *crs1* deletion mutant, this reduction is the expected one for an additive effect (1–0.72x0.55 = 0.60), indicating these CDK-complexes might control DSB formation acting in genetically independent pathways (Fig 3). In spite of this reduction in DSBs, NCOs and COs are not correspondingly affected, and the levels in the double mutants are similar to those observed in the single mutant *crs1* (Fig 1B and 1C). A possible explanation for this discrepancy is that in the absence of Cig1 repair is biased towards homologous chromatids and not sister chromatids, which in fission yeast is the most common template used (1:3 proportion) [87]. This observation could imply that Cdc2-Cig1 contributes to crossover invariance, a phenomenon that suggests differential partner choice for repair at hotspots (with the sister chromatid) and coldspots (with a homologous chromatid) to maintain a constant chromosome CO distribution in spite of different frequency of DSB formation across the genome [39]. Alternatively, the reduction in DSBs may be locus-dependent in the double *cig1 crs1* deletion mutant.

The requirement of cyclins for meiotic recombination has been recently studied, and Cig1, Cig2, and Crs1 reported not to have a role in the process [9]. In the case of Cig1 and Cig2, recombination was exclusively addressed in intergenic recombination assays, and wild-type levels of COs reported. Accordingly, we have not found reduction in CO levels in these mutants; however, DSBs and NCOs are reduced indicating that indeed these CDK-complexes regulate meiotic recombination. Additional roles of these cyclins in steps downstream of DSB formation may obscure the outcome of the intergenic recombination assays. In the case of Crs1, both NCOs and COs were genetically analyzed in the published work, and normal levels reported in the *crs1* mutant. However, we have consistently found statistically significant DSB, NCO, and CO reductions in this mutant (complete ORF deletion, see Material and Methods), even in recombination assays using the same published genetic intervals (Figs 1 and 3). We do not have an explanation for this discrepancy apart from possible differences in genetic backgrounds that, although well known in budding yeast to influence both mitotic and meiotic phenotypes [88–91] are not well documented in fission yeast laboratory strains [92].

The role of CDK activity in DSB formation is supported by our results using controlled chemical inhibition of the *cdc2-asM17* allele [74]. DSB formation is significantly impaired, both at hotspots in the NotI J fragment and genome-wide, when CDK activity is inhibited prior to meiotic induction or after DNA replication when cells are entering into prophase (Figs 4 and 5). The fact that DSB formation is not restored when the replication checkpoint is abrogated using a *rad3* deletion mutant indicates that this inhibition of DSB formation is not an indirect consequence of checkpoint activation (S5 Fig). Finally, the fact that in *crs1* and *cig1 crs1* mutants DSBs are reduced meanwhile meiotic progression is normal (S4 Fig), suggests that CDK activity drives DSB formation directly and not indirectly by promoting meiotic progression. The observation of DSB formation when the ATP-analog is partially inactivated in the experiments with the *cdc2-asM17* allele strengthens this view; meiotic progression is completely blocked in this situation (no chromosome segregations), however break formation is reactivated (Fig 5 and S5 Fig).

**Fig 10:**
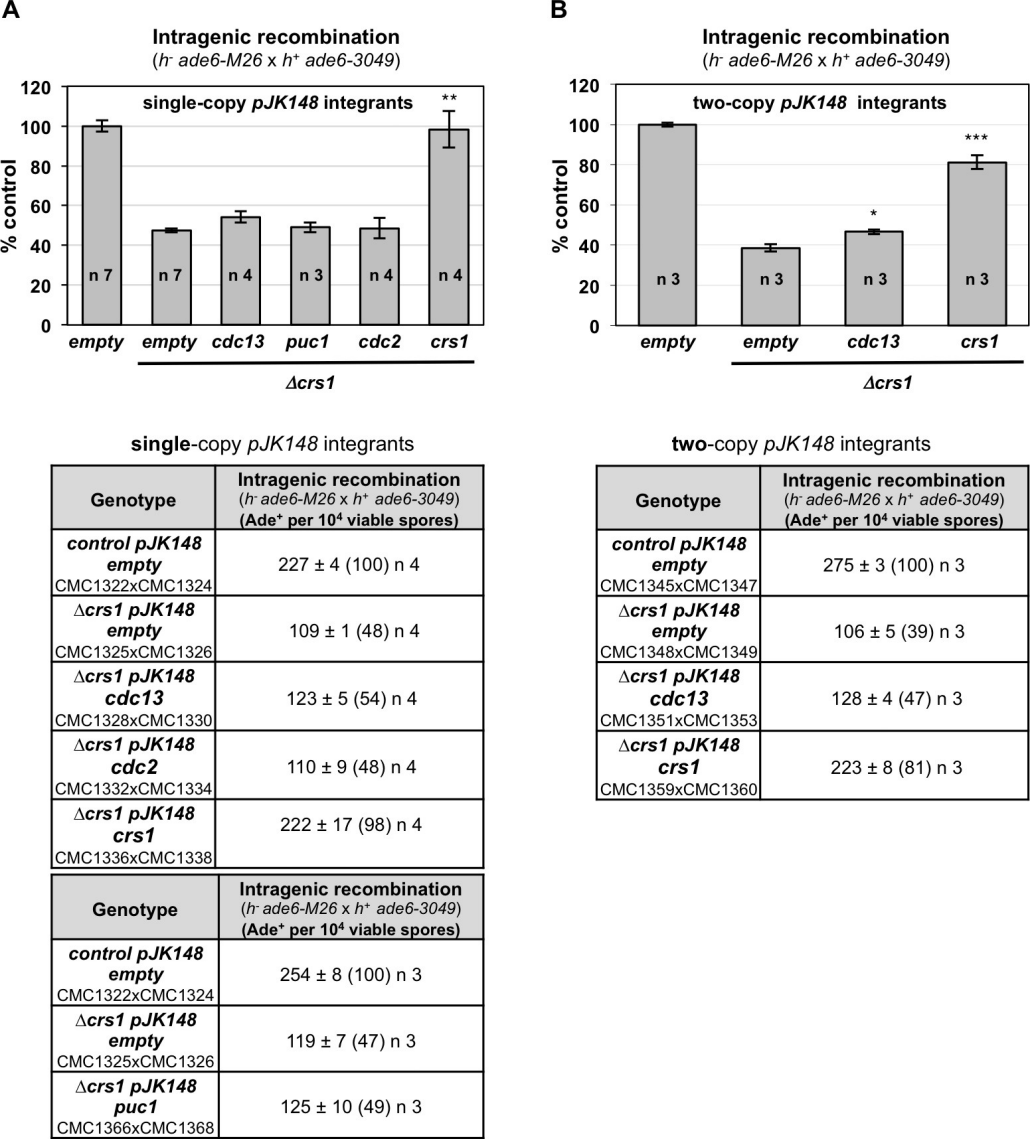
Increasing copy number of *cdc13*, *puc1*, or *cdc2* does not restore recombination levels in *Δcrs1*. Crosses of *h*^*-*^ *ade6-M26* x *h*^*+*^ *ade6-3049* were performed in SPA and plated for recombinant frequency at least twice. Tables at the bottom show gene conversion expressed as the mean of Ade^+^ per 10^4^ viable spores +/- SEM of n independent crosses based on the cumulative number of spore colonies in each cross; 71–293 Ade^+^ colonies scored in each independent cross. The numbers in parentheses are percentages relative to the control integrant (*pJK148 empty*). Strains used in the crosses are indicated. Graphs at the top show gene conversion expressed as mean of the percentage relative to the control cross +/- SEM of n independent crosses. Each integrant was analyzed only with its control crosses (*pJK148 empty* and *Δcrs1 pJK148 empty*) in the same experiment. *p* values were calculated based on Student´s t-test (unpaired, two tails), * <0.05, ** <0.01, *** <0.001. **(A)** Single-copy integrants. **(B)** Two-copy integrants.

The stronger reduction in DSBs in these experiments compared to the levels detected in single and double cyclin deletion mutants indicates that other CDK complexes may contribute to break formation. Redundancy of cyclins has been extensively reported in fission yeast where only Cdc13 is essential [8, 9]. Therefore, even cyclins normally not abundant during meiotic prophase such as Puc1 and Rem1 could contribute to DSB formation in the absence of Cig1, Cig2, and Crs1. Additionally, Cdc13 is by large the cyclin that contributes the most to the total cellular CDK activity, and the increasing levels of Cdc13 during prophase may also regulate DSB formation. A role of Puc1 in recombination has been suggested since, in contrast to the double *cig1 cig2* deletion mutant, a triple *cig1 cig2 puc1* mutant reduces crossover levels [9]. In addition, overexpression of a Cdc13-Cdc2 fusion protein, as unique source of CDK activity in the cell, partially sustains recombination; interestingly, the same fusion protein Cdc13-Cdc2 efficiently restores meiotic progression [9]. We have evaluated possible compensatory/redundant effects by increasing copy number of *cdc13* and *puc1* cyclins in *crs1* mutants. Increasing genomic copies of *cdc13* or *puc1* cyclins (even two-copy insertion in the case of *cdc13*) does not restore the recombination defect of *crs1* deletion mutants. Moreover, neither increasing genomic copies of *cdc2* (which presumably would increase levels of the different CDK complexes) restores it (Fig 10). These data suggest Crs1 specificity to promote meiotic recombination.

Cdc2 implication in DSB formation was previously studied in synchronous haploid *pat1-114* meiosis using a temperature-sensitive *cdc2* allele, and DSB formation was detected but not quantified [50]. It is possible that Cdc2 was not completely inhibited in those experiments, and/or the ploidy of the cell could make a difference. Ploidy of the cell has an impact on the requirement of the replication checkpoint for the meiotic arrest upon hydroxyurea (HU) treatment, and diploid cells are more dependent than haploid cells on the Rad3/Cds1 pathway to block meiotic progression [93–95]. Similarly, DSB formation could be more sensitive to the inhibition of CDK activity in diploid than haploid meiosis. However, in both cases the inhibition of DSB formation upon HU treatment strongly depends on the Rad3/Cds1 pathway [53, 56], indicating that, if the different Cdc2-requirement for DSB formation were due to ploidy of the cell, the Rad3/Cds1 pathway would not account for this difference. Interestingly, untreated *cdc2-asM17* mutants progress through meiotic S-phase faster than wild-type cells, although meiosis I entry is not advanced, indicating a prophase extension (S7 Fig). Nevertheless, despite the fact that the cells finish replication earlier, DSB formation is not advanced (or extended) compared to the timing of DSB appearance and disappearance in a wild-type strain (compare control in Fig 2 and Fig 3 to control in Fig 4 and Fig 5). This observation suggests that although S-phase progression and DSB formation are coordinated (see Introduction), S-phase completion is not sufficient for the activation of break formation.

We have found that LinE formation is impaired when CDK activity is modulated by depleting Cig1 and Crs1 cyclins (Fig 6 and S6 Fig), or the global CDK activity is reduced by using the *cdc2-asM17* allele and an inhibitor (Fig 9B). In both cases, a defect in LinE maturation is observed with Rec25-GFP. Although the kinetics of signal accumulation is normal, mature signal is reduced at least 2.5–3 fold compared to the controls (Figs 6 and 9B). Since nuclear foci formation of any of the LinE components depends on each of the others [15, 16], we infer that the abnormal maturation of the Rec25-GFP signal reflects a defect in LinE maturation. Moreover, this defect is even observed in the *cdc2-asM17* allele without treatment (Fig 8B). This cytological defect may be related to the reduced chromatin-binding capability of Rec25-GFP, since in *cig1 crs1* deletion mutants, *cdc2-asM17* without treatment, and *cdc2-asM17* after CDK inhibition, the protein is more easily recovered from the nuclear fraction by detergent treatment (Fig 7). A more labile binding of Rec25-GFP (and the LinE complex) to the meiotic chromosomes could impair proper LinE organization and subsequent DSB formation, since these complexes are essential for DSB formation in most hotspots [15]. CDK control of synaptonemal complex (SC) formation has been reported in budding yeast where the spreading of the central-element protein Zip1 depends on Cdc28 activity; however, no molecular explanation for this phenotype was described [96].

We have generated phospho-null mutants in putative CDK phosphorylation sites of Rec10 and Rec27 (the only LinE-components harboring CDK sites), Rec7 (SFT-complex), and Rec14 (DSBC-complex) proteins (S8 Fig). None of the mutants tested (alone or in different combinations) reduces levels of gene conversion (in quantitative or qualitative assays), except a moderate 20% reduction in *rec14 (cdk1)* mutants (S9 Fig and S10 Fig). Although we have not exploited all the possible mutants and combinations, so far our mutational analysis suggests that there is not a clear main CDK substrate to control DSB formation in fission yeast. It is possible that the effect we describe in LinE and DSB formation is indirectly mediated by mis-regulation of cohesins. Meiotic cohesins are required for LinE formation [14–16, 19–21], and meiotic cohesin subunits Rec8 and Rec11 harbor putative CDK phosphorylation sites, some of them phosphorylated *in vivo* [22, 97, 98]. Finally, at least Cdc2-Cig2 activity controls Mei4-promotor occupancy to promote timely activation of the middle wave of meiotic transcriptional-induction [99], including *mde2* expression. Mde2 has been proposed to stabilize SFT and DSBC-complex interaction [14]. Therefore, appropriate timing in the formation/loading of pre-recombination complexes may be an additional mechanism to optimize DSB formation. It is possible that in fission yeast several CDK-regulated targets (in different processes) equally contribute to DSB formation and recombination, and we may need cumulative defects in this regulation in order to observe a defect in recombination.

Our results show that CDK activity regulates the initiation of meiotic recombination, namely DSB formation, in fission yeast. Maturation of LinEs (essential for DSB formation at hotspots) seems to be an important point of CDK regulation, modulating the binding to chromatin of one of their structural components, Rec25. Though CDK activity has been implicated in the biology of the meiotic chromosomes and recombination in different organisms [100–106], a role in DSB formation was clearly established previously only in *Saccharomyces cerevisiae*. Given the evolutionary distance between budding and fission yeasts, our work strengths this view and points to CDK regulation of DSB formation as a conserved feature of meiotic recombination. In addition, the comparison between DSB levels and recombination outcomes (NCOs and COs) suggests additional points of CDK regulation downstream of break formation, balancing NCOs/COs and intersister/interhomolog repair. Finally, among the cyclins analyzed, meiosis-specific Crs1 shows the major contribution to DSB formation, and complementation analysis suggests specificity for this cyclin to promote recombination.

## Materials and methods

### Yeast manipulation and general methods

Experimentally required strains were obtained by meiotic crosses. Strains used in this study are listed in S1 Table. Oligos used are listed in S2 Table. Cells were grown in yeast extract medium with supplements (YES) or Edinburgh minimal medium (MM) with supplements at 32°C or 25°C (for temperature-sensitive mutants). Normal supplements were Adenine, Leucine, Uracil and Histidine (225 mg/l). YES supplemented with 0.1 mg/ml G-418 (Formedium) or Hygromycin B (Formedium) was used to select and follow deletion mutants and GFP-tagged gene versions. Genetics crosses were done in malt extract plates with supplements (MEA-4S) at 25°C. Diploid *pat1-114 leu1-32* strains were obtained by protoplast fusion and selection for complementation of *ade6-M210* and *ade6-M216* alleles [107]. Synchronous meiosis by thermal inactivation at 34°C of the *pat1-114* temperature-sensitive allele and cell collection for flow cytometry analysis were done as previously described [16]. When experimentally required 20 μM 1-NM-PP1 (Toronto Research Chemicals Inc.) or equal DMSO volume was added to the cultures. A Becton Dickinson FACSCalibur and CellQuest software were used for cell acquisition and data analysis. Cellular fractionations were done as previously described [108], and equal extract equivalent of each fraction (7% of Whole Cell Extract) analyzed by Western blot with primary anti-GFP (monoclonal JL-8, Living colors, Clontech), anti-α-Tubulin (monoclonal Clone B-5-1-2, Sigma) and anti-Histone H4 (rabbit polyclonal ab10158, Abcam) antibodies; and secondary anti-mouse light chain-specific (115-035-174 Jackson ImmunoResearch) and anti-rabbit (A6154 Sigma) horseradish peroxidase-conjugated antibodies. Rec25-GFP signal was developed with SuperSignal West DURA extended Kit (Pierce), and Tubulin and Histone H4 signals with ECL Western Blotting Kit (Amersham, GE Healthcare). Rec25-GFP quantification was done with Image J 1.49b software (NIH) and under-saturated scan exposures (ChemiDoc XRS Imaging System, Bio-Rad). Microscopy used to detect Rec25-GFP signal in Methanol/Acetone fixed cells was previously described [15]. Images of whole cells are maximal projections of 11 sections at 0.4 μm steps to cover the whole cell (4 μm total). DNA images are a single focal plane, because out-of-focus DAPI fluorescence obscures the projection. Cytological classification of the different Rec25-GFP categories was done using the raw images and navigating the Z sections. Images were captured with a Nikon Eclipse 90i microscope equipped with a 100x/NA1.45/WD0.13 Oil Plan APO Lambda lens, a Hamamatsu ORCA-ER camera, and MetaMorph software (Molecular Devices).

### Construction of deletion, integrants, and point mutants

New *cig1* and *crs1* deletions were generated. Available *cig1* deletion [109] removes 363 bp of adjacent ORF (*rec11*), coding for a meiosis-specific cohesin subunit already known to be important for meiotic recombination [15, 17]. In the case of *crs1*, a change in ORF annotation has extended previous ORF designation and the original deletion maintains 20% of the ORF [65]. Complete *cig1* and *crs1* ORF deletions were generated by PCR-based method [110] using oligos to amplify *hphMX6* from plasmid pFA6a-hphMX6 and transformation to Hygromycin B resistance of strain CMC6 (*h*^*90*^ *ura4-D18*) in the case of *cig1*, and CMC66 (*h*^*-*^ *pat1-114 ade6-M210 leu1-32 rec25-GFP*::*KanMX6*) in the case of *crs1*. These oligos were pair cig1-D1/cig1-D2 and pair crs1-D1/crs1-D2.

pJK148 *cdc13* (4.2 kb genomic clone containing 1869 bp upstream and 865 bp downstream of the ORF) and pIRT22 *cdc2* (3.4 kb genomic clone containing 840 bp upstream and 1372 bp downstream of the ORF) plasmids were a gift from Sergio Moreno´s laboratory. *cdc2* was subcloned in the pJK148 plasmid at the PstI restriction site. *puc1* (3.9 kb fragment containing 2360 bp upstream and 485 bp downstream of the ORF) and *crs1* (2.5 kb fragment containing 1083 bp upstream and 513 bp downstream of the ORF) genomic clones were PCR amplified using oligo pairs puc1-SacI/puc1-KpnI and crs1-SalI(3)/crs1-EcoRI(3). All pJK148 plasmids were sequenced. For integration plasmids were digested with NdeI (pJK148 *cdc2*), NruI (pJK148 *empty* and pJK148 *cdc13*) or Tth111I (pJK148 *crs1* and pJK148 *puc1*), and the strain CMC1056 (*h*^*-*^ *crs1*::*hphMX6 leu1-32*) transformed to Leu^+^. Single copy and multicopy integrants were selected by PCR using oligos pJK148-1/pJK148-2; and specific plasmid integrations tested using oligo pairs pJK148-upKpnI/pJK148-downBamHI (pJK148 *empty*), pJK148-upKpnI/cdc13-1 (pJK148 *cdc13*), pJK148-upKpnI/cdc2-10 (pJK148 *cdc2*), pJK148-upKpnI/puc1-2 (pJK148 *puc1*), and pJK148-upKpnI/crs1-3 (pJK148 *crs1*). Number of *leu1* copies in multicopy integrants was checked by qPCR using the oligo pairs leu1-3/leu1-4 and mde2-3/mde2-4 (internal control); the parental CMC1056 strain and a single copy integrant were used as controls. pJK148 *puc1* and pJK148 *cdc2* multicopy integrants were found to be reorganized.

*rec7 (cdk1*, *cdk2*, *cdk1 cdk2)*, *rec14 (cdk1*, *cdk2*, *cdk1 cdk2)*, and *rec27* (*cdk*) mutants in putative CDK phosphorylation sites were generated by PCR using plasmids pFA6a-rec7-GFP-hphMX6 (CMC28, containing 191 bp upstream ORF + ORF), pFA6a-rec14-GFP-kanMX6 (CMC43, containing 152 bp upstream ORF + ORF), and pFA6a-rec27-GFP-hphMX6 (CMC34, containing 184 bp upstream ORF + ORF) as templates, and the following oligo pairs: rec7-cdk1F/rec7-cdk1R, rec7-cdk2F/rec7-cdk2R, rec14-cdk1F/rec14-cdk1R, rec14-cdk2F/rec14-cdk2R, and rec27-cdkF/rec27-cdkR. PCR products were digested with DpnI and transformed into *Escherichia coli*, plasmids recovered and sequenced. Double *cdk1 cdk2* mutants were similarly generated using the plasmids containing single mutant genes as templates. Cassettes for *S*. *pombe* transformation were obtained by PCR using as templates the plasmids containing the different mutants and the following oligo pairs: rec7-3/rec7-STOP (which amplify unmarked and untagged versions), rec14-1/rec14-D2 (which amplify G-418 resistant GFP-versions) or rec14-1/rec14-STOP (which amplify unmarked and untagged versions), and rec27-1/rec27-D2 (which amplify Hygromycin B resistant GFP-versions) or rec27-1/rec27-STOP (which amplify unmarked and untagged versions). *rec7* cassettes were used for transformation of strain CMC945 (*h*^*-*^ *rec7*::*ura4*^*+*^ *ura4-D18*) to FOA resistance. *rec14* cassettes were used for transformation of strain CMC595 (*h*^*90*^ *rec14*::*hphMX6*) to G-418 resistance or strain CMC1201 (*h*^*-*^ *rec14*::*ura4*^*+*^ *ura4-D18*) to FOA resistance. *rec27* cassettes were used for transformation of strain CMC952 (*h*^*90*^ *rec27*::*kanMX6*) to Hygromycin B resistance or strain CMC966 (*h*^*-*^ *rec27*::*ura4*^*+*^ *ura4-D18*) to FOA resistance. *rec10 (cdk total)* and *rec14* (*cdk total*) mutants were synthetic fragments (Integrated DNA Technologies) that were PCR amplified and transformed into CMC1201 (*h*^*-*^ *rec14*::*ura4*^*+*^ *ura4-D18*) and CMC1218 (*h*^*-*^ *rec10*::*ura4*^*+*^ *ura4-D18*) strains. *rec10 (cdk total)* harbors an extra mutation (C597 to A) changing F199 (TTC codon) to L (TTA codon). Deletion strains *rec7*::*ura4*^*+*^, *rec10*::*ura4*^*+*^, *rec14*::*ura4*^*+*^, *rec14*::*hphMX6*, and *rec27*::*ura4*^*+*^ used for knock-ins were done by PCR-based method [110] using oligos to amplify *ura4* gene or *hphMX6* from plasmids pFA6a-ura4 and pFA6a-hphMX6 respectively, and transformation to Ura^+^ prototrophy or Hygromycin B resistance of strains CMC4 (*h*^*-*^ *ura4-D18*) and CMC3 (*h*^*90*^ 968). Oligo pairs used for these deletions were: rec7-D1/rec7-D2, rec10-D1/rec10-D2, rec14-D1/rec14-D2, and rec27-D1/rec27-D2. Correct deletions and knock-ins were checked by PCR and sequencing.

### Recombination assays

Crosses were done in MEA-4S or SPA at 25°C. After 3–4 days cell masses were treated overnight at 25°C with glucuronidase (Roche), and subsequently incubated 25 minutes at 55°C to kill any remaining vegetative cells. For intragenic recombination assays the *ade6-M26* allele was always in the *h*^*-*^ parent, and the *ade6-3049* or *ade6-M210* allele in the *h*^*+*^ parent. For intergenic recombination assays the *leu1-32* marker was always in the *h*^*-*^ parent, and the *his5-303* marker in the *h*^*+*^ parent. For intragenic recombination assays appropriate numbers of viable spores were plated on 10 YE-minus supplement plates (approx. 600/plate) or 10 YE+Guanine plates (10^4^/plate; Guanine inhibits Adenine uptake and kills Ade^-^ cells [111, 112]), and incubated for 4–5 days at 32°C. Frequency of intragenic recombination was calculated as the number of white colonies (Ade^+^ in YE) per 10^4^ viable spores, pooling the numbers of the 10 plates. Each experiment was plated twice and the final recombination frequency was calculated based on cumulative numbers of the two platings. For frequency of intergenic recombination 300–500 viable spores were plated on 5 YES plates, and after 3 days at 32°C replicated to YES-Phloxin B (to identify diploid colonies and discard for further analysis) and MM (to score for Leu^+^ His^+^ colonies). Frequency of intergenic recombination was calculated as the number of haploid prototrophic colonies (Leu^+^ His^+^) per 100 haploid spore colonies. In the case of *mat1-P* and *leu1-32* markers, spore colonies grown at 32°C in 5 YES plates were replicated to YES-Phloxin B (to discard diploids) and MM (to score for Leu^+^), and the number of haploid prototrophic Leu^+^ and total colonies scored. 240 Leu^+^ colonies were randomly selected and grown as patched in YES plates. These master plates were further replicated to 2 new YES plates. Next day patches in one of the plates were individually mixed with a dense *h*^*+*^ cell suspension, and further incubated at 32°C for one day. Then, both plates were replicated to MEA-4S, and after 6–7 days at 25°C exposed to iodine vapors to determine the presence of spores (indicative of mating). The plate with patches previously not mixed with *h*^*+*^ cells was used to discard *h*^*90*^ (*h*^*+*^ revertants) colonies from the scoring. Frequency of intergenic recombination was calculated as the number of haploid *h*^*-*^ prototrophic colonies (iodine-positive Leu^+^) per 100 haploid spore colonies, scaling the number of *h*^*-*^ Leu^+^ colonies in the sample (240 selected Leu^+^ colonies) to the number of total Leu^+^ colonies, and to the number of total colonies scored. As for intragenic recombination, each experiment for intergenic recombination was plated twice and the final recombination frequency calculated based on cumulative numbers of the two platings. Recombination assays were repeated 3–10 times and *p* values were calculated based on Student´s t-test (unpaired, two tails).

### PFGE and DSB quantification

For detection of DSBs Pulse Field Gel Electrophoresis (PFGE) of agarose embedded samples (plugs) was used. 30 ml cell samples (O. D. 0.8–1) at different times during meiotic time courses were processed as described in [113] with some modifications. Collected cells were washed with 30 ml of cold 50 mM EDTA pH 8.0 and resuspended in 300 μl of cold CEPES (50 mM EDTA pH 8.0, 40 mM Na_2_HPO_4_, 20 mM citric acid, 1.2 M sorbitol, 10 mM sodium azide, 1mg/ml Zymolyase 20T from *Arthrobactor luteus* (Seikagaku Biobusiness Corporation) and 5 mg/ml lysing enzymes from *Trichoderma harzianum* (Sigma)). Samples were kept on ice until collection of the last time-point samples, and then all were processed in parallel. Cells were incubated at 37°C for 1.5 hr in a thermoblock with gentle agitation. After checking for proper cell wall digestion, all samples were put on ice and agarose plugs then prepared. Samples were warmed at 50°C for 1 min in a thermoblock, mixed with 400 μl of low melting-point agarose (1% agarose in 50 mM EDTA pH 8.0, 10 mM Tris-HCl pH 7.5, 1.2 M sorbitol) at 50°C, and divided into the plug molds. Molds were cooled at 4°C for 15 min to solidify, ejected into 2 ml Eppendorf tubes containing 1.2 ml of 0.25 M EDTA pH 8.0, 50 mM Tris-HCl pH 7.5, 1% SDS, and incubated at 50°C for 90 min. Afterwards, solution was replaced by 1.2 ml of Lysis Buffer (0.5 M EDTA pH 8.0, 10 mM Tris-HCl pH 7.5, 10 mM sodium azide, 1% N-Lauroylsarcorine sodium) with 1mg/ml Proteinase K (Roche) and plugs incubated overnight at 50°C. Next day, Lysis Buffer was replaced with fresh Lysis Buffer with Proteinase K, and plugs incubated at 50°C until next day. Finally, Proteinase K was inactivated washing the plugs in 1.2 ml of TE (10 mM Tris-HCl pH 8, 1 mM EDTA) with 1 mM PMSF for 2 hr at room temperature, and three times in TE for 30 min-1 hr at room temperature with gentle agitation prior to final store at 4°C. For detection of DSBs in intact chromosomes plugs were washed with 500 μl of TAE (40 mM Tris-Acetate, 1 mM EDTA pH 8) for 1 hr with gentle agitation at room temperature prior to loading in a 0.7% agarose (Pulsed Field Certified Megabase Agarose, Bio-Rad) TAE gel. Gels were run in a CHEF-DR II system (Bio-Rad) for 70h at 2V/cm, 30 min of both initial and final switch time, 120° angle, and 14°C. Finally, gels were stained overnight at room temperature in TAE with 0.5 μg/ml of Ethidium Bromide. For detection of DSBs at hotspot *mbs1* by Southern blot, plugs were washed twice for 30 min at 4°C in 250 μl of enzyme buffer, buffer replaced by 250 μl of fresh buffer containing 35U of NotI, and incubated at 4°C during 6–7 hr before final incubation at 37°C overnight. Next day, plugs were washed in 1 ml of 0.5X TBE (90mM Tris, 90 mM Boric Acid, 2 mM EDTA, pH 8.3) for 1 hr with gentle agitation at room temperature prior to loading in a 1.1% agarose (Pulsed Field Certified Megabase Agarose) 0.5X TBE gel. Gels were run for 24h at 6V/cm, 7.9 seconds of initial switch and 54.2 seconds of final switch, 120° angle, and 14°C. After electrophoresis, gels were stained with 0.5 μg/ml of Ethidium Bromide for 30 min to check proper digestion prior to Southern blotting. Vacuum (Vacugen, Amersham) or capillarity alkaline transfer to Nylon membranes (Amersham Hybond-XL, GE Healthcare) was performed before standard Southern blot [114] using a ^32^P radiolabelled probe recognizing the left end of the 501 Kb NotI fragment J [72]. DSB quantification was done with Quantity One software (Bio-Rad) and under-saturated phosphorimager exposures (PMI Personal Molecular Images, Bio-Rad; Fuji imaging BAS-III screens). Counts (CNT x mm) in the whole lane for each sample and at *mbs1* position were obtained, after background lane elimination using the Rolling Disk (10) function. Whole lane was considered from top gel excluding the wells to the bottom just below the *mbs2* site. Correction factor for the difference in total DNA between samples before and after DNA replication was calculated by dividing the total signal of the sample with maximal levels of DSBs (after DNA replication) by the total signal of the nitrogen-depleted sample (before DNA replication). The specific signal at the *mbs1* band in the nitrogen-depleted sample was multiplied by this factor, and correspondingly subtracted from the *mbs1* signal of the rest of the time points of the experiment. Corrected *mbs1* signals were then divided by the total lane signal to obtain the percentage of breakage at each time point. Assays were repeated 5–8 times and *p* values were calculated based on Student´s t-test (unpaired, two tails).

## Supporting information

S1 Fig Spore viability of different cyclin deletion mutants. The same crosses as for the gene conversion assays in Fig 1 (*h*^*-*^ *ade6-M26* x *h*^*+*^ *ade6-3049*) were performed in MEA and spores plated twice in YES. Graph shows spore viability expressed as mean of the percentage relative to the control cross +/- SEM of 4 independent crosses based on the cumulative number of spore colonies in each cross; 2414–5568 colonies scored in each independent cross. The spore viability of the mutants is not significantly different from the wild-type control cross, except for the *cig1 cig2* double mutant (* *p* value 0.035). *p* values were calculated based on Student´s t-test (unpaired, two tails).
(TIF)

S2 Fig Analysis of meiotic progression in *cig1*, *cig2*, and *cig1 cig2* deletion mutants. **(A)** Flow cytometry analysis of synchronous diploid *pat1-114* meiosis of control (CMC7), *cig1* (CMC1010), *cig2* (CMC1022), and double *cig1 cig2* (CMC1023) deletion mutants. DNA content (FL2-H) and cell size (FSC) histograms are shown. Dashed-lined box outlines premeiotic S-phase progression. **(B)** Quantification of chromosome segregation by DAPI staining and nuclear counting (1 nucleus, 2 nuclei, 3 nuclei, and 4 nuclei) is shown. The arrows indicate meiosis I (MI) entry, and the vertical dashed-lines indicate the peak of MI.
(TIF)

S3 Fig DSB formation is reduced in *crs1* mutants (deficient DSB-repair condition, *rad50S*). **(A)** Detection of *mbs1* breakage by Southern blot in control (CMC967) and *crs1* deletion mutants (CMC1177) during synchronous meiosis of *pat1-114 rad50S* diploids. Percentage of breakage is represented on the right. **(B)** PFGE separation of entire chromosomes during the same meiotic kinetics. DSBs are visualized as a cumulative smear below the chromosomes due to unrepaired breakage in *rad50S* [72]. Control and *crs1* mutant were analyzed in the same gel and therefore similarly stained and subjected to the same image processing.
(TIF)

S4 Fig Analysis of meiotic progression in *cig1*, *crs1*, and *cig1 crs1* deletion mutants. **(A)** Flow cytometry analysis of synchronous diploid *pat1-114* meiosis of control (CMC7), *cig1* (CMC1010), *crs1* (CMC1059), and double *cig1 crs1* (CMC1113) deletion mutants. DNA content (FL2-H) and cell size (FSC) histograms are shown. Dashed-lined box outlines premeiotic S-phase progression. **(B)** Quantification of chromosome segregation by DAPI staining and nuclear counting (1 nucleus, 2 nuclei, 3 nuclei, and 4 nuclei) is shown. The arrows indicate meiosis I (MI) entry, and the vertical dashed-lines indicate the peak of MI.
(TIF)

S5 Fig Chemical inhibition of Cdc2 blocks DSB formation in *rad3* mutants (CDK inhibition after DNA replication). **(A) On the left** Flow cytometry analysis of synchronous haploid *pat1-114 cdc2-asM17 rad3* meiosis (CMC1165) with DMSO (top left panel) or 20 μM 1-NM-PP1 (top right panel) added at 2 hr 15 min (arrow in the bottom panel) after meiotic induction of a common culture. DNA content (FL2-H) histograms are shown. **On the right** Quantification of chromosome segregation by DAPI staining and nuclear counting (1 nucleus, 2 nuclei, ≥ 3 nuclei) is shown. Timing of meiosis I (MI) and meiosis II (MII) is indicated. Arrows indicate time of DMSO or 1-NM-PP1 addition. **(B) Top panel** Detection of *mbs1* breakage by Southern blot during the same meiotic kinetics. Double-headed arrows indicate temporal position (2.5–4 hr) of DSB formation in the control. Percentage of breakage is indicated on top; u undetectable (<0.1%). Partial inactivation of the ATP-analog at later time points (4 hr) allows DSB formation without chromosome segregation. Similar result (significant inhibition of DSB formation at *mbs1* hotspot) was obtained in an independent experiment adding the ATP-analog at the beginning of the kinetics. **Bottom panel** PFGE separation of entire chromosomes during the same meiotic kinetics. DSBs are visualized as a transient smear below the chromosomes. Double-headed arrows indicate temporal position (2.5–4 hr) of DSB formation in the control. Similar result (genome-wide significant inhibition of DSB formation) was obtained in an independent experiment adding the ATP-analog at the beginning of the kinetics.
(TIF)

S6 Fig Defective LinE maturation in double *cig1 crs1* deletion mutants. Synchronous diploid *pat1-114 rec25-GFP* meiosis of control (CMC78) and double *cig1 crs1* deletion mutants (CMC1207) were induced. **(A)** Meiotic progression. **On the top** Flow cytometry analysis showing DNA content (FL2-H) histograms. Dashed-lined box outlines premeiotic S-phase progression. **On the bottom** Quantification of chromosome segregation by DAPI staining and nuclear counting (1 nucleus, 2 nuclei, 3 nuclei, and 4 nuclei) is shown. The arrows indicate meiosis I (MI) entry, and the vertical dashed-lines indicate the peak of MI. **(B)** Rec25-GFP localization. Photographs of cells (Methanol/Acetone fixed) at different times during prophase are shown. Rec25-GFP (left panels), and merged Rec25-GFP and DNA (DAPI-staining) (right panels). Labeled cells in the control time course correspond to the different categories quantified in this study: diffuse nuclear signal (stars), diffuse nuclear+foci signal (circles), and mature signal (arrows). Notice the presence of cells with “diffuse nuclear+foci” signal at late time points in the double *cig1 crs1* deletion mutant. Bottom images in the *cig1 crs1* experiment show cells with tangled signal (arrowheads), representing at 3.5 hr 27% of the population compared to 4% in the control kinetics.
(TIF)

S7 Fig Untreated *cdc2-asM17* mutants show a faster S-phase progression. **(A)** Flow cytometry analysis of synchronous diploid *pat1-114* control (CMC7) and *pat1-114 cdc2-asM17* (CMC1066) meiosis. Neither DMSO nor ATP-analog was added to the cells. DNA content (FL2-H) histograms are shown. **(B)** Quantification of chromosome segregation by DAPI staining and nuclear counting (1 nucleus, 2 nuclei, 3 nuclei, and 4 nuclei) is shown. The arrows indicate meiosis I (MI) entry, and the vertical dashed-lines indicate the peak of MI.
(TIF)

S8 Fig Proteins required for DSB formation. Essential proteins for DSB formation at meiotic hostpots are listed. Proteins are grouped based on the complexes they belong to (LinE, SFT, and DSBC-complexes). Names in parenthesis correspond to *S*. *cerevisiae* orthologs. Number and position of minimal and consensus CDK sites present in these proteins are indicated. Names in red correspond to the proteins subject to mutational studies. On the right column names of the different generated mutants for the selected proteins are annotated.
(TIF)

S9 Fig Recombination assays with *rec7*, *rec14*, *rec10*, and *rec27* mutants in putative CDK phosphorylation sites. Crosses of *h*^*-*^ *ade6-M26* x *h*^*+*^ *ade6-3049* homozygous for the different mutants were performed in MEA and plated for recombinant frequency at least twice. Tables on the right show gene conversion expressed as the mean of Ade^+^ per 10^4^ viable spores +/- SEM of n independent crosses based on the cumulative number of spore colonies in each cross; 73–345 Ade^+^ colonies scored in each independent cross. The numbers in parentheses are percentages relative to the corresponding control. Strains used in the crosses are indicated. Graphs on the left show gene conversion expressed as mean of the percentage relative to the control cross +/- SEM of the same n independent crosses. Not statistically significant differences based on Student´s t-test (unpaired, two tails).
(TIF)

S10 Fig Qualitative recombination assays with single, double, and triple *rec7*, *rec14* and *rec27* mutants in putative CDK phosphorylation sites. Crosses of *h*^*-*^ *ade6-M26* x *h*^*+*^ *ade6-3049* homozygous for the different mutants were performed in MEA and an equal number of spores (10^4^ and serial 1/4 dilutions) plotted on YEA, YE+Guanine, MM+Adenine, and MM plates. A cross with a *rec14-GFP* version was used as a control of the sensitivity of the assay since this GFP-tagged Rec14 protein supports approx. 65% of the wild-type recombination efficiency (S9 Fig). The mutants used alone or in different combinations were *rec7 (cdk1 cdk2)*, *rec14 (cdk1 cdk2)*, and *rec27 (cdk)*, all of them unmarked knock-ins. Strains used in the crosses are indicated.
(TIF)

S1 Table *S*. *pombe* strains. Alleles other than commonly used auxotrophies and mating type are *ade6-3049* [115], *ade6-M26* [116], *pat1-114* [73], *rad50S* [72], *cig2*::*ura4*^*+*^ [117], *rec25-204*::*GFP-kanMX6* [Rec25-GFP] [16], *rec27-184*::*kanMX6* [65], *cdc2-asM17* [74], and *rad3*::*ura4*^+^ [118, 119].
*cig1*::*hphMX6*, *crs1*::*hphMX6*, *rec7*::*ura4*^*+*^, *rec7-cdk1*, *rec7-cdk2*, *rec7-cdk1cdk2*, *rec10*::*ura4*^*+*^, *rec10-cdk total*, *rec14*::*ura4*^*+*^, *rec14*::*hphMX6*, *rec14-GFP*::*kanMX6*, *rec14-cdk1-GFP*::*kanMX6*, *rec14-cdk2-GFP*::*kanMX6*, *rec14-cdk1cdk2-GFP*::*kanMX6*, *rec14-cdk1cdk2*, *rec14-cdk total*, *rec27*::*ura4*^*+*^, *rec27-GFP*::*hphMX6*, *rec27-cdk-GFP*::*hphMX6*, and *rec27-cdk* alleles were generated in this study (Material and Methods).
(DOCX)

S2 Table Oligonucleotides. (DOCX)
