# Supplementary figures and images for: CDK contribution to DSB formation and recombination in fission yeast meiosis
Source: PLoS Genet. 2019 Jan 14;15(1):e1007876. doi: 10.1371/journal.pgen.1007876 (PMC6331086; doi:10.1371/journal.pgen.1007876)

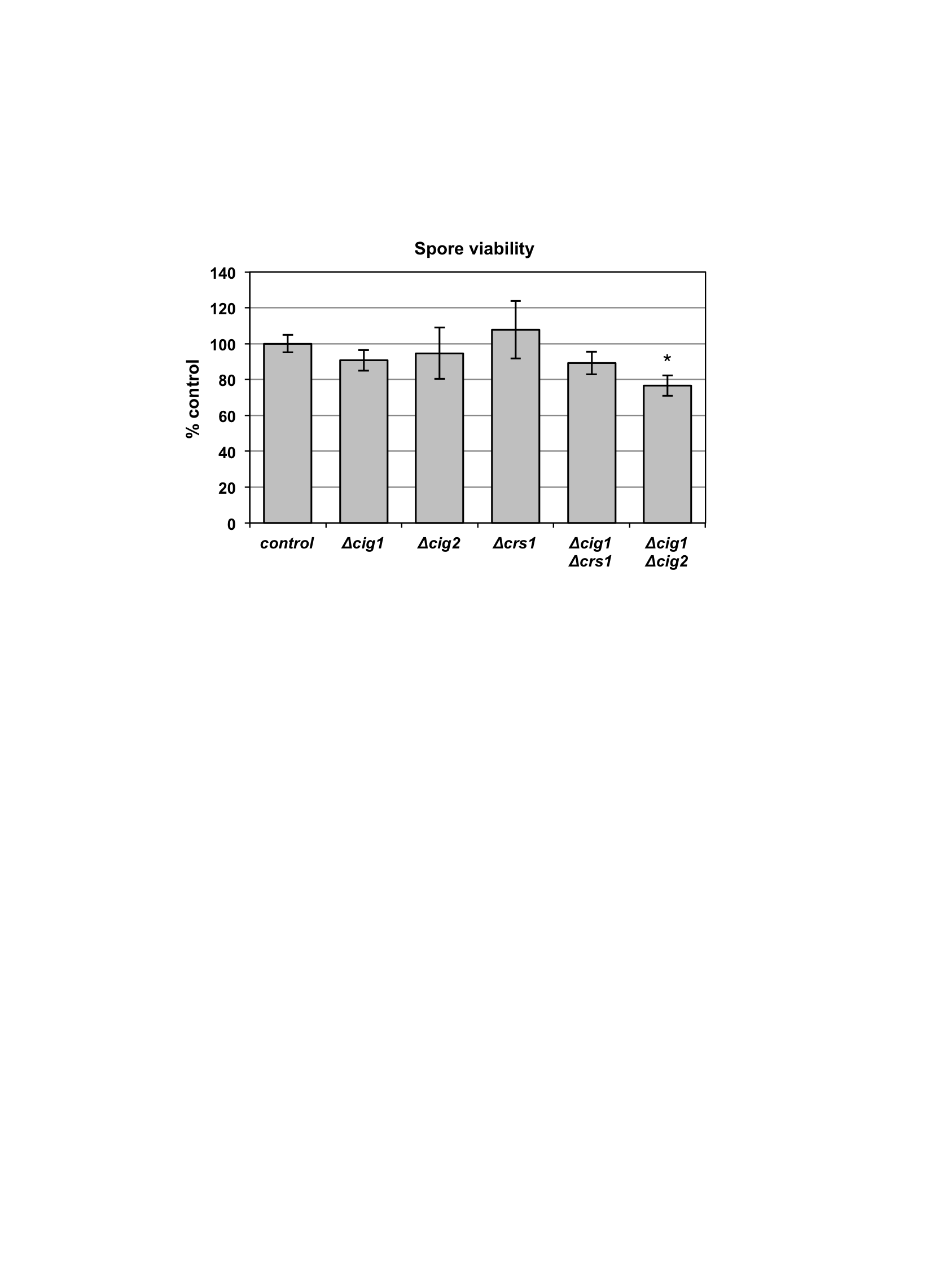

Supplement: S1 Fig — The same crosses as for the gene conversion assays in Fig 1 (h- ade6-M26 x h+ ade6-3049) were performed in MEA and spores plated twice in YES. Graph shows spore viability expressed as mean of the percentage relative to the control cross +/- SEM of 4 independent crosses based on the cumulative number of spore colonies in each cross; 2414–5568 colonies scored in each independent cross. The spore viability of the mutants is not significantly different from the wild-type control cross, except for the cig1 cig2 double mutant (* p value 0.035). p values were calculated based on Student´s t-test (unpaired, two tails). (TIF) [file pgen.1007876.s001.tif]

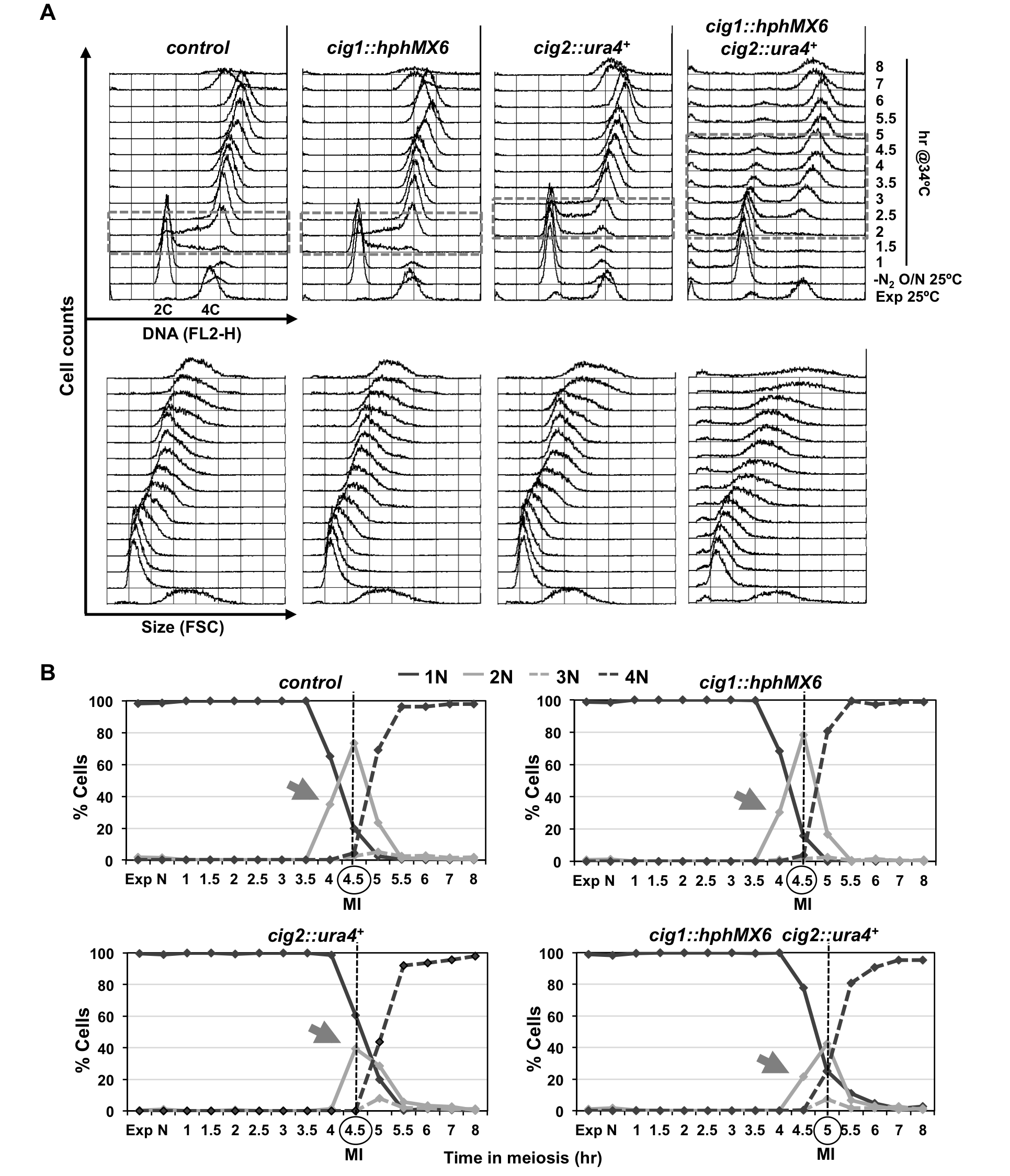

Supplement: S2 Fig — (A) Flow cytometry analysis of synchronous diploid pat1-114 meiosis of control (CMC7), cig1 (CMC1010), cig2 (CMC1022), and double cig1 cig2 (CMC1023) deletion mutants. DNA content (FL2-H) and cell size (FSC) histograms are shown. Dashed-lined box outlines premeiotic S-phase progression. (B) Quantification of chromosome segregation by DAPI staining and nuclear counting (1 nucleus, 2 nuclei, 3 nuclei, and 4 nuclei) is shown. The arrows indicate meiosis I (MI) entry, and the vertical dashed-lines indicate the peak of MI. (TIF) [file pgen.1007876.s002.tif]

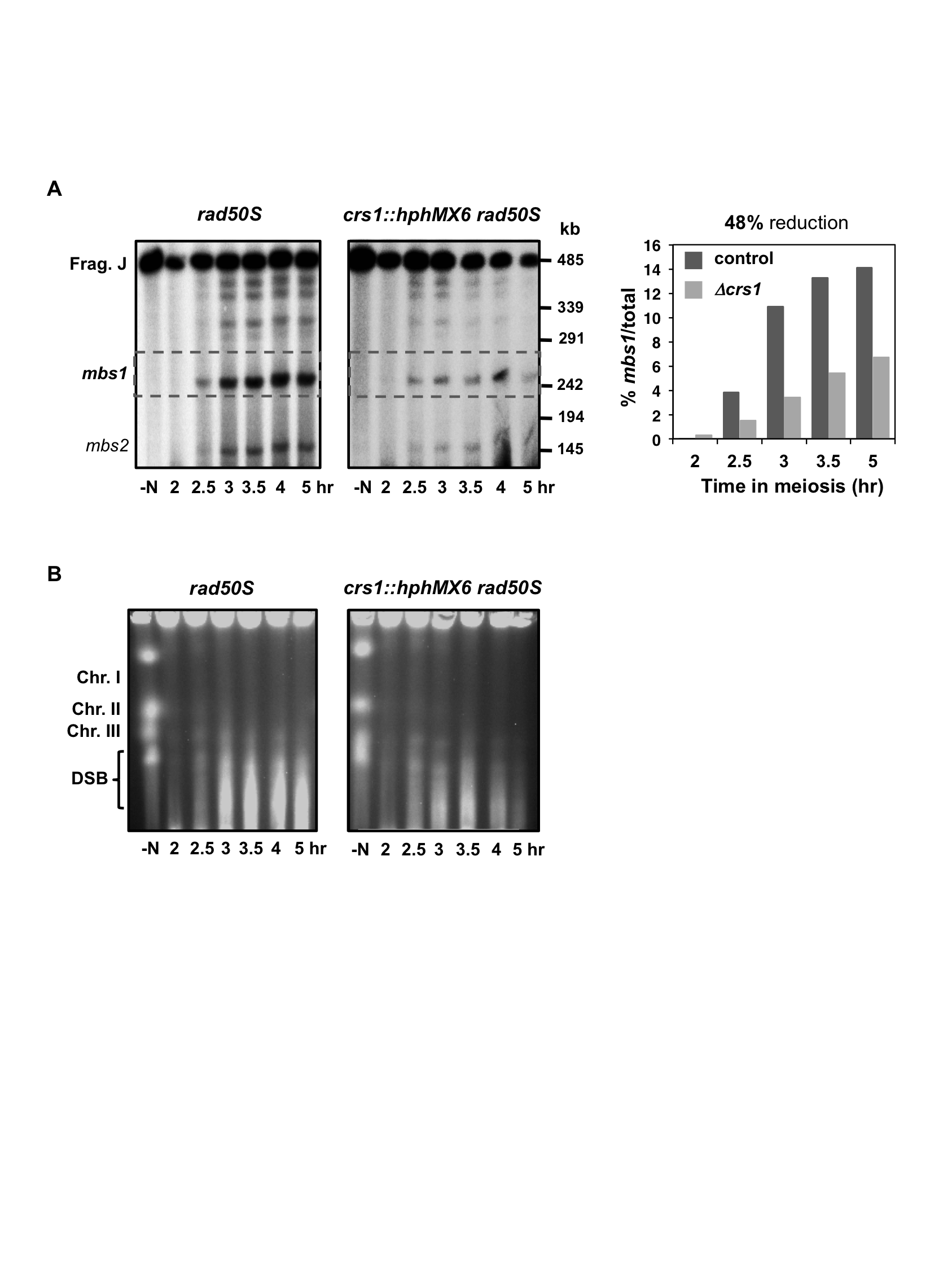

Supplement: S3 Fig — (A) Detection of mbs1 breakage by Southern blot in control (CMC967) and crs1 deletion mutants (CMC1177) during synchronous meiosis of pat1-114 rad50S diploids. Percentage of breakage is represented on the right. (B) PFGE separation of entire chromosomes during the same meiotic kinetics. DSBs are visualized as a cumulative smear below the chromosomes due to unrepaired breakage in rad50S [72]. Control and crs1 mutant were analyzed in the same gel and therefore similarly stained and subjected to the same image processing. (TIF) [file pgen.1007876.s003.tif]

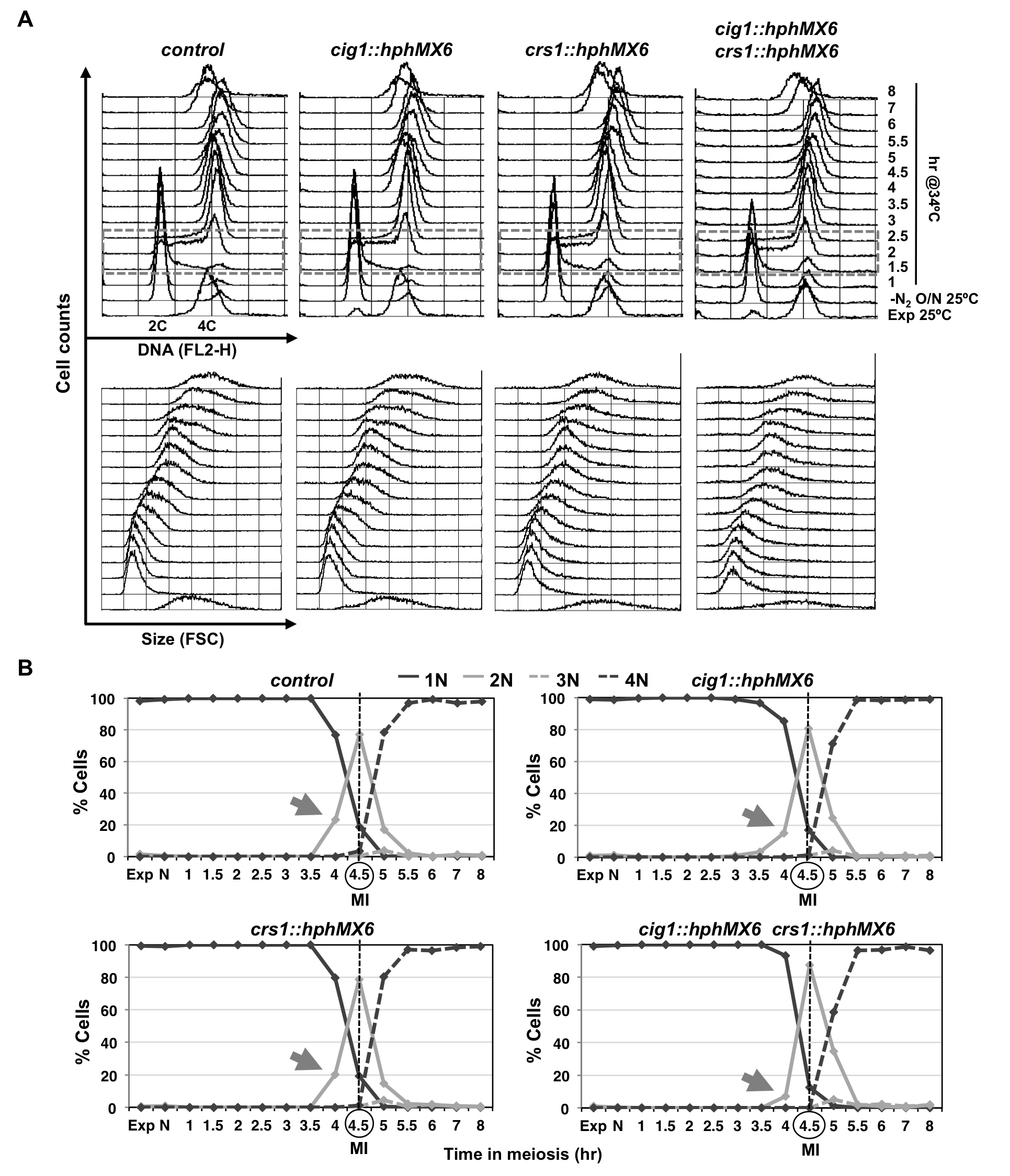

Supplement: S4 Fig — (A) Flow cytometry analysis of synchronous diploid pat1-114 meiosis of control (CMC7), cig1 (CMC1010), crs1 (CMC1059), and double cig1 crs1 (CMC1113) deletion mutants. DNA content (FL2-H) and cell size (FSC) histograms are shown. Dashed-lined box outlines premeiotic S-phase progression. (B) Quantification of chromosome segregation by DAPI staining and nuclear counting (1 nucleus, 2 nuclei, 3 nuclei, and 4 nuclei) is shown. The arrows indicate meiosis I (MI) entry, and the vertical dashed-lines indicate the peak of MI. (TIF) [file pgen.1007876.s004.tif]

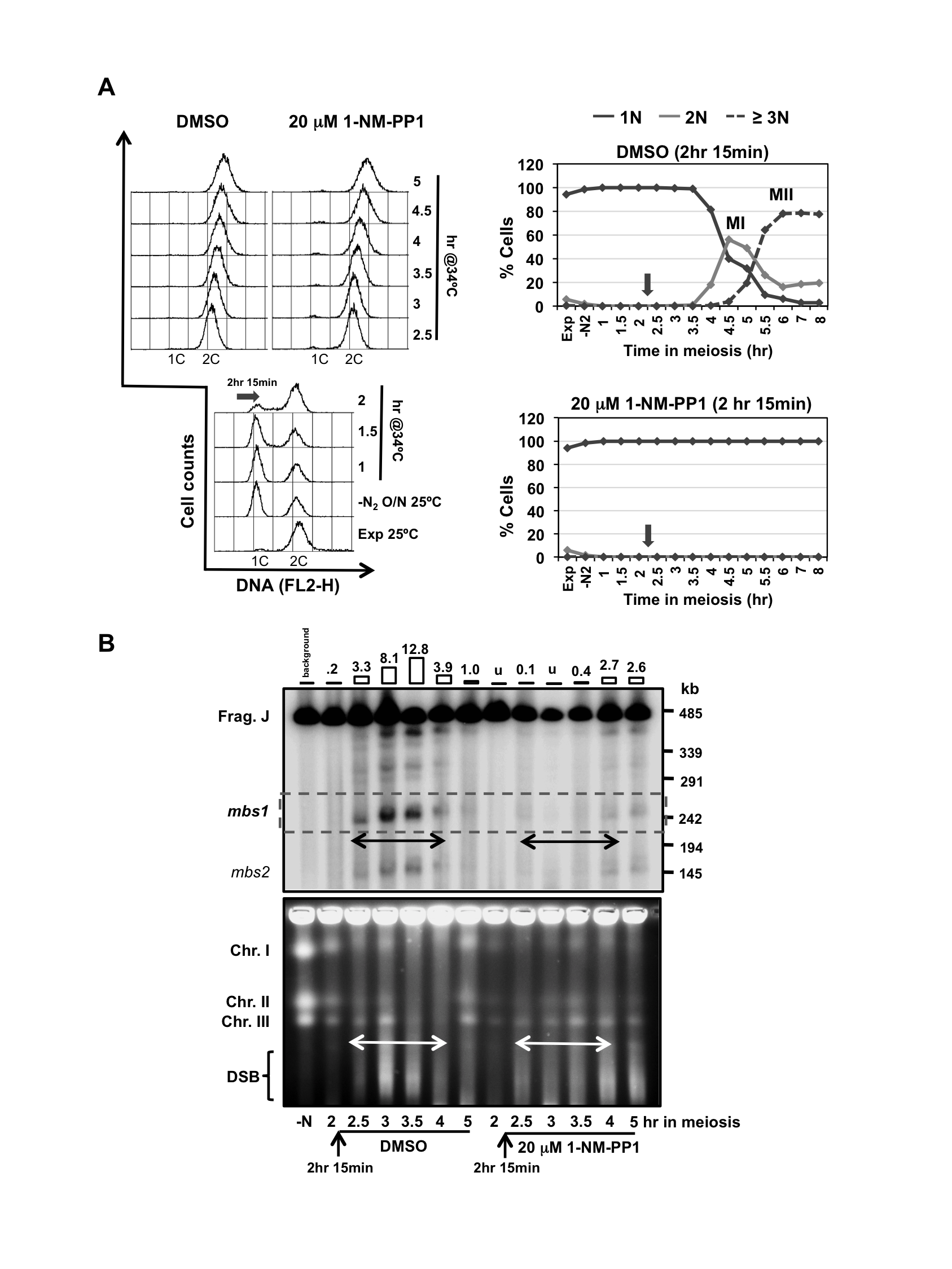

Supplement: S5 Fig — (A) On the left Flow cytometry analysis of synchronous haploid pat1-114 cdc2-asM17 rad3 meiosis (CMC1165) with DMSO (top left panel) or 20 μM 1-NM-PP1 (top right panel) added at 2 hr 15 min (arrow in the bottom panel) after meiotic induction of a common culture. DNA content (FL2-H) histograms are shown. On the right Quantification of chromosome segregation by DAPI staining and nuclear counting (1 nucleus, 2 nuclei, ≥ 3 nuclei) is shown. Timing of meiosis I (MI) and meiosis II (MII) is indicated. Arrows indicate time of DMSO or 1-NM-PP1 addition. (B) Top panel Detection of mbs1 breakage by Southern blot during the same meiotic kinetics. Double-headed arrows indicate temporal position (2.5–4 hr) of DSB formation in the control. Percentage of breakage is indicated on top; u undetectable (<0.1%). Partial inactivation of the ATP-analog at later time points (4 hr) allows DSB formation without chromosome segregation. Similar result (significant inhibition of DSB formation at mbs1 hotspot) was obtained in an independent experiment adding the ATP-analog at the beginning of the kinetics. Bottom panel PFGE separation of entire chromosomes during the same meiotic kinetics. DSBs are visualized as a transient smear below the chromosomes. Double-headed arrows indicate temporal position (2.5–4 hr) of DSB formation in the control. Similar result (genome-wide significant inhibition of DSB formation) was obtained in an independent experiment adding the ATP-analog at the beginning of the kinetics. (TIF) [file pgen.1007876.s005.tif]

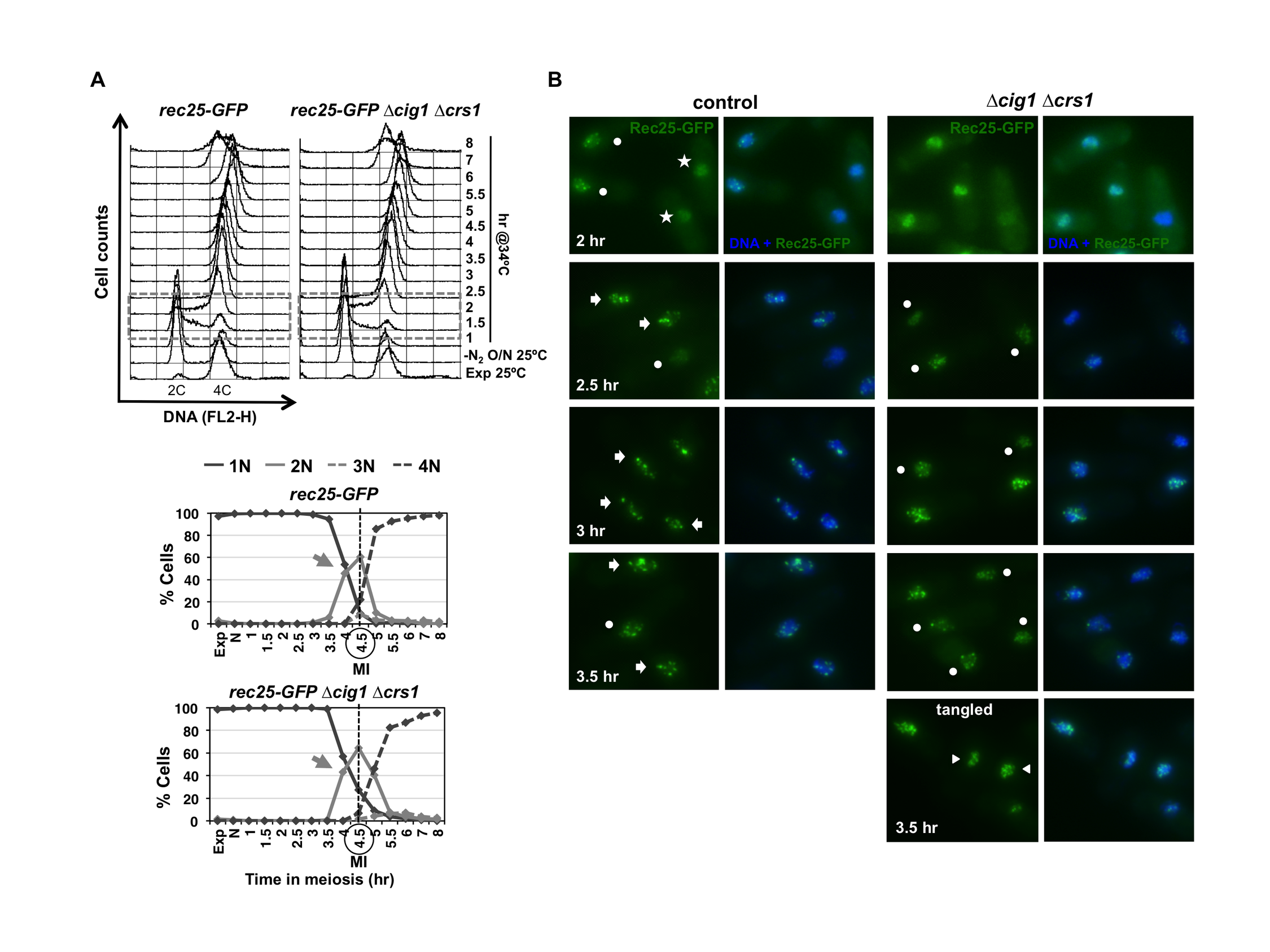

Supplement: S6 Fig — Synchronous diploid pat1-114 rec25-GFP meiosis of control (CMC78) and double cig1 crs1 deletion mutants (CMC1207) were induced. (A) Meiotic progression. On the top Flow cytometry analysis showing DNA content (FL2-H) histograms. Dashed-lined box outlines premeiotic S-phase progression. On the bottom Quantification of chromosome segregation by DAPI staining and nuclear counting (1 nucleus, 2 nuclei, 3 nuclei, and 4 nuclei) is shown. The arrows indicate meiosis I (MI) entry, and the vertical dashed-lines indicate the peak of MI. (B) Rec25-GFP localization. Photographs of cells (Methanol/Acetone fixed) at different times during prophase are shown. Rec25-GFP (left panels), and merged Rec25-GFP and DNA (DAPI-staining) (right panels). Labeled cells in the control time course correspond to the different categories quantified in this study: diffuse nuclear signal (stars), diffuse nuclear+foci signal (circles), and mature signal (arrows). Notice the presence of cells with “diffuse nuclear+foci” signal at late time points in the double cig1 crs1 deletion mutant. Bottom images in the cig1 crs1 experiment show cells with tangled signal (arrowheads), representing at 3.5 hr 27% of the population compared to 4% in the control kinetics. (TIF) [file pgen.1007876.s006.tif]

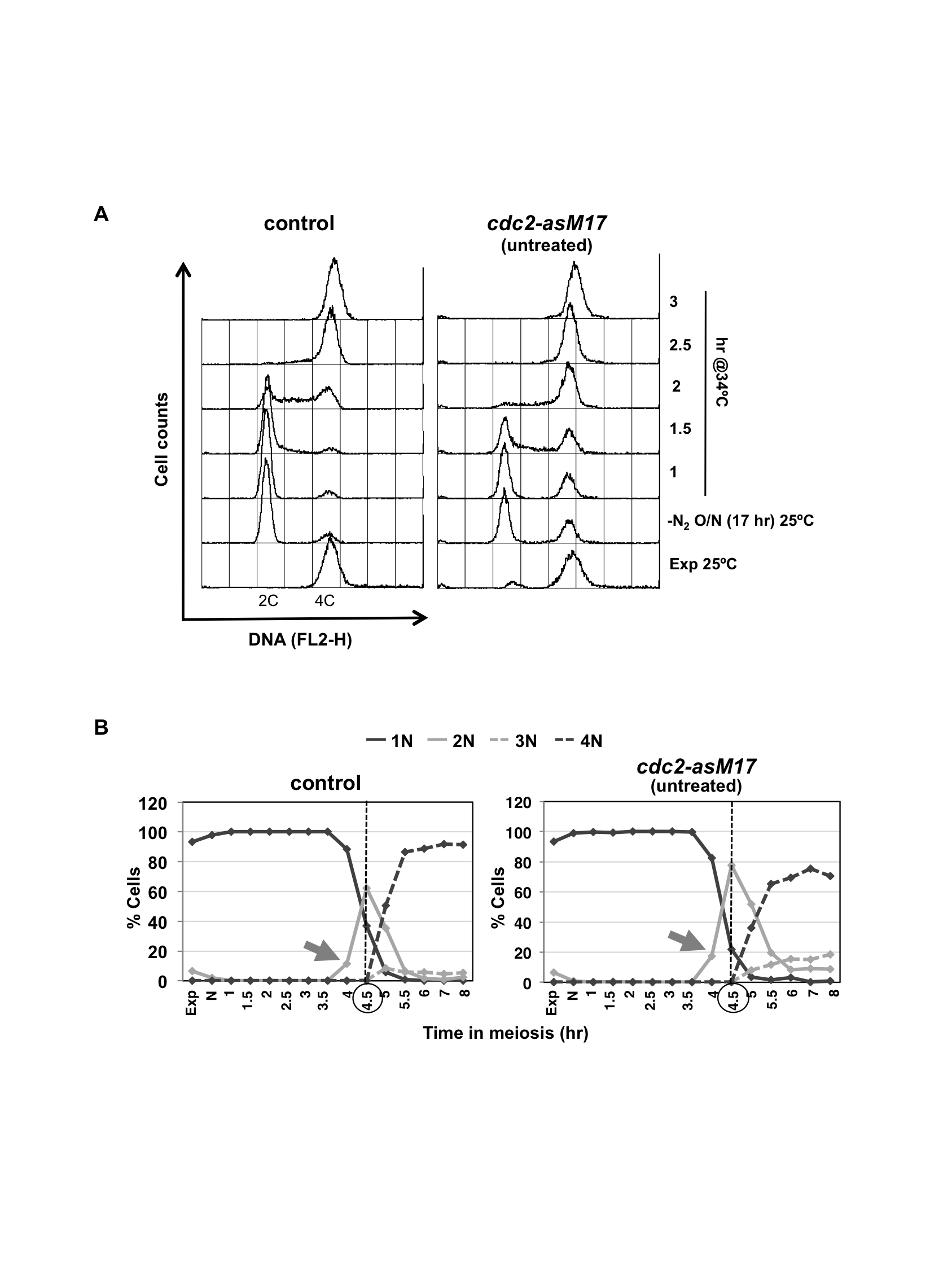

Supplement: S7 Fig — (A) Flow cytometry analysis of synchronous diploid pat1-114 control (CMC7) and pat1-114 cdc2-asM17 (CMC1066) meiosis. Neither DMSO nor ATP-analog was added to the cells. DNA content (FL2-H) histograms are shown. (B) Quantification of chromosome segregation by DAPI staining and nuclear counting (1 nucleus, 2 nuclei, 3 nuclei, and 4 nuclei) is shown. The arrows indicate meiosis I (MI) entry, and the vertical dashed-lines indicate the peak of MI. (TIF) [file pgen.1007876.s007.tif]

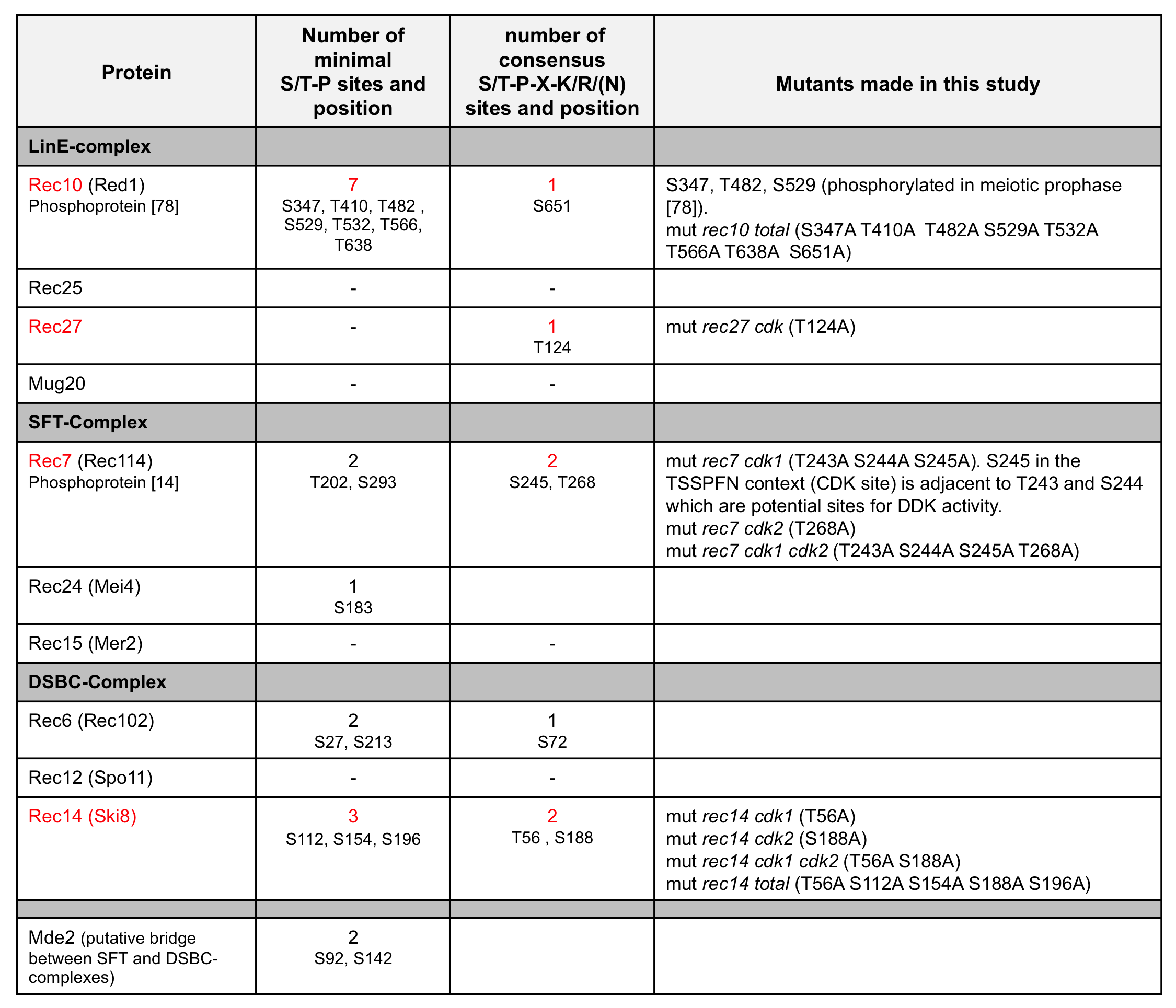

Supplement: S8 Fig — Essential proteins for DSB formation at meiotic hostpots are listed. Proteins are grouped based on the complexes they belong to (LinE, SFT, and DSBC-complexes). Names in parenthesis correspond to S. cerevisiae orthologs. Number and position of minimal and consensus CDK sites present in these proteins are indicated. Names in red correspond to the proteins subject to mutational studies. On the right column names of the different generated mutants for the selected proteins are annotated. (TIF) [file pgen.1007876.s008.tif]

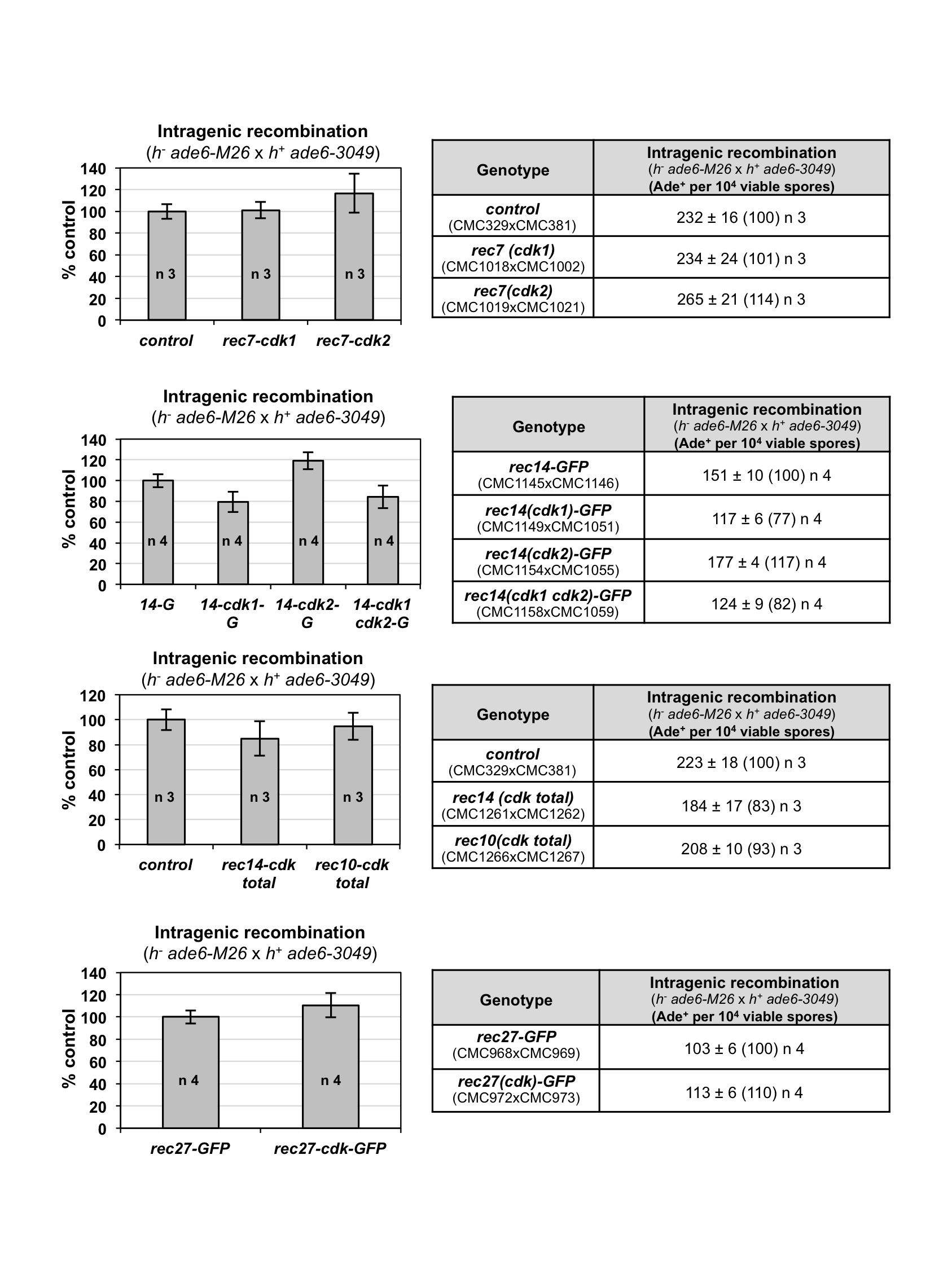

Supplement: S9 Fig — Crosses of h- ade6-M26 x h+ ade6-3049 homozygous for the different mutants were performed in MEA and plated for recombinant frequency at least twice. Tables on the right show gene conversion expressed as the mean of Ade+ per 104 viable spores +/- SEM of n independent crosses based on the cumulative number of spore colonies in each cross; 73–345 Ade+ colonies scored in each independent cross. The numbers in parentheses are percentages relative to the corresponding control. Strains used in the crosses are indicated. Graphs on the left show gene conversion expressed as mean of the percentage relative to the control cross +/- SEM of the same n independent crosses. Not statistically significant differences based on Student´s t-test (unpaired, two tails). (TIF) [file pgen.1007876.s009.tif]

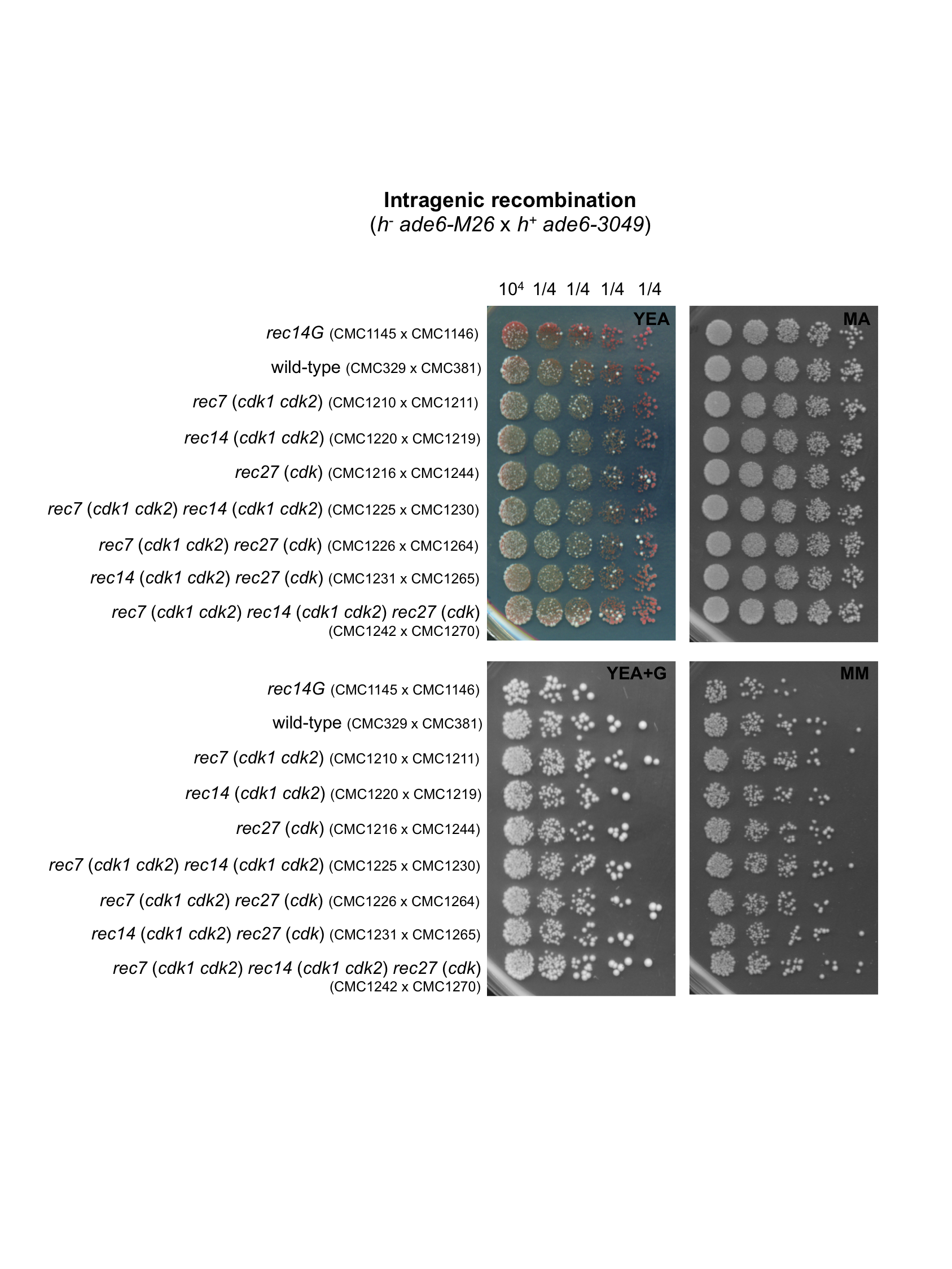

Supplement: S10 Fig — Crosses of h- ade6-M26 x h+ ade6-3049 homozygous for the different mutants were performed in MEA and an equal number of spores (104 and serial 1/4 dilutions) plotted on YEA, YE+Guanine, MM+Adenine, and MM plates. A cross with a rec14-GFP version was used as a control of the sensitivity of the assay since this GFP-tagged Rec14 protein supports approx. 65% of the wild-type recombination efficiency (S9 Fig). The mutants used alone or in different combinations were rec7 (cdk1 cdk2), rec14 (cdk1 cdk2), and rec27 (cdk), all of them unmarked knock-ins. Strains used in the crosses are indicated. (TIF) [file pgen.1007876.s010.tif]
