# Supplementary material for: CDK contribution to DSB formation and recombination in fission yeast meiosis
Source: PLoS Genet. 2019 Jan 14;15(1):e1007876. doi: 10.1371/journal.pgen.1007876 (PMC6331086; doi:10.1371/journal.pgen.1007876)
Supplement: S1 Table — Alleles other than commonly used auxotrophies and mating type are ade6-3049 [115], ade6-M26 [116], pat1-114 [73], rad50S [72], cig2::ura4+ [117], rec25-204::GFP-kanMX6 [Rec25-GFP] [16], rec27-184::kanMX6 [65], cdc2-asM17 [74], and rad3::ura4+ [118, 119]. cig1::hphMX6, crs1::hphMX6, rec7::ura4+, rec7-cdk1, rec7-cdk2, rec7-cdk1cdk2, rec10::ura4+, rec10-cdk total, rec14::ura4+, rec14::hphMX6, rec14-GFP::kanMX6, rec14-cdk1-GFP::kanMX6, rec14-cdk2-GFP::kanMX6, rec14-cdk1cdk2-GFP::kanMX6, rec14-cdk1cdk2, rec14-cdk total, rec27::ura4+, rec27-GFP::hphMX6, rec27-cdk-GFP::hphMX6, and rec27-cdk alleles were generated in this study (Material and Methods). (DOCX) [file pgen.1007876.s011.docx]

| **Strain** | **Genotype** | **Used in** |
| --- | --- | --- |
| **CMC2** | *h^+^975* | Fig 1 |
| **CMC7** | *h^-^/h^-^ pat1-114/pat1-114 ade6-M210/ade6-M216 leu1-32/leu1-32* | Fig 2, 3, S2, and S4 |
| **CMC78** | *h^-^/h^-^ pat1-114/pat1-114 ade6-M210/ade6-M216 leu1-32/leu1-32 rec25-GFP::kanMX6/rec25-GFP::kanMX6* | Fig 6, 7, 8, and S6 |
| **CMC319** | *h^-^ leu1-32* | Fig 1 |
| **CMC329** | *h^-^ ade6-M26* | Fig 1, S1, S9, and S10 |
| **CMC381** | *h^+^ade6-3049* | Fig 1, S1, S9, and S10 |
| **CMC570** | *h^+^ ade6-3049 cig2::ura4^+^ ura4-D18* | Fig 1 and S1 |
| **CMC571** | *h^-^ ade6-M26 cig2::ura4^+^ ura4-D18* | Fig 1 and S1 |
| **CMC612** | *h^+^ ade6-3049 cig1::hphMX6 cig2::ura4^+^ ura4-D18* | Fig 1 and S1 |
| **CMC613** | *h^-^ ade6-M26 cig1::hphMX6 cig2::ura4^+^ ura4-D18* | Fig 1 and S1 |
| **CMC616** | *h^+^ ade6-3049 cig1::hphMX6* | Fig 1 and S1 |
| **CMC617** | *h^-^ ade6-M26 cig1::hphMX6* | Fig 1 and S1 |
| **CMC735** | *h^+^ his5-303 cig1::hphMX6* | Fig 1 |
| **CMC737** | *h^+^ his5-303* | Fig 1 |
| **CMC741** | *h^-^ leu1-32 cig1::hphMX6* | Fig 1 |
| **CMC889** | *h^+^ ade6-M210* | Fig 1 |
| **CMC967** | *h^-^/h^-^ pat1-114/pat1-114 ade6-M210/ade6-M216 leu1-32/leu1-32 rad50S/rad50S* | Fig S3 |
| **CMC968** | *h^-^ ade6-M26 rec27-GFP::hphMX6* | Fig S9 |
| **CMC969** | *h^+^ ade6-3049 rec27-GFP::hphMX6* | Fig S9 |
| **CMC972** | *h^-^ ade6-M26 rec27-cdk-GFP::hphMX6* | Fig S9 |
| **CMC973** | *h^+^ ade6-3049 rec27-cdk-GFP::hphMX6* | Fig S9 |
| **CMC1002** | *h^+^ ade6-3049 rec7-cdk1* | Fig S9 |
| **CMC1010** | *h^-^/h^-^ pat1-114/pat1-114 ade6-M210/ade6-M216 leu1-32/leu1-32 cig1::hphMX6/cig1::hphMX6* | Fig 2, 3, S2, and S4 |
| **CMC1018** | *h^-^ ade6-M26 rec7-cdk1* | Fig S9 |
| **CMC1019** | *h^-^ ade6-M26 rec7-cdk2* | Fig S9 |
| **CMC1021** | *h^+^ ade6-3049 rec7-cdk2* | Fig S9 |
| **CMC1022** | *h^-^/h^-^ pat1-114/pat1-114 ade6-M210/ade6-M216 leu1-32/leu1-32 ura4-D18/ura4-D18 cig2::ura4^+^/cig2::ura4^+^* | Fig 2 and S2 |
| **CMC1023** | *h^-^/h^-^ pat1-114/pat1-114 ade6-M210/ade6-M216 leu1-32/leu1-32 ura4-D18/ura4-D18 cig2::ura4^+^/cig2::ura4^+^ cig1::hphMX6/cig1::hphMX6* | Fig 2 and S2 |
| **CMC1052** | *h^-^ ade6-M26 crs1::hphMX6* | Fig 1 and S1 |
| **CMC1054** | *h^+^ ade6-3049 crs1::hphMX6* | Fig 1 and S1 |
| **CMC1056** | *h^-^ leu1-32 crs1::hphMX6* | Fig 1 |
| **CMC1058** | *h^+^ his5-303 crs1::hphMX6* | Fig 1 |
| **CMC1059** | *h^-^/h^-^ pat1-114/pat1-114 ade6-M210/ade6-M216 leu1-32/leu1-32 crs1::hphMX6/crs1::hphMX6* | Fig 3 and S4 |
| **CMC1066** | *h^-^/h^-^ pat1-114/pat1-114 ade6-M210/ade6-M216 leu1-32/leu1-32 cdc2-asM17/cdc2-asM17* | Fig. 4, 5, and S7 |
| **CMC1086** | *h^+^ ade6-3049 crs1::hphMX6 cig1::hphMX6* | Fig 1 and S1 |
| **CMC1088** | *h^-^ ade6-M26 crs1::hphMX6 cig1::hphMX6* | Fig 1 and S1 |
| **CMC1113** | *h^-^/h^-^ pat1-114/pat1-114 ade6-M210/ade6-M216 leu1-32/leu1-32 crs1::hphMX6/crs1::hphMX6 cig1::hphMX6/cig1::hphMX6* | Fig 3 and S4 |
| **CMC1114** | *h^-^ leu1-32 crs1::hphMX6 cig1::hphMX6* | Fig 1 |
| **CMC1115** | *h^+^ his5-303 crs1::hphMX6 cig1::hphMX6* | Fig 1 |
| **CMC1118** | *h^+^ ade6-M210 crs1::hphMX6* | Fig 1 |
| **CMC1119** | *h^+^ crs1::hphMX6* | Fig 1 |
| **CMC1121** | *h^-^ leu1-32 cig2::ura4^+^ ura4-D18* | Fig 1 |
| **CMC1132** | *h^+^ his5-303 cig2::ura4^+^ ura4-D18* | Fig 1 |
| **CMC1145** | *h^-^ ade6-M26 rec14-GFP::KanMX6* | Fig S9 and S10 |
| **CMC1146** | *h^+^ ade6-3049 rec14-GFP::KanMX6* | Fig S9 and S10 |
| **CMC1149** | *h^-^ ade6-M26 rec14-cdk1-GFP::KanMX6* | Fig S9 |
| **CMC1151** | *h^+^ ade6-3049 rec14-cdk1-GFP::KanMX6* | Fig S9 |
| **CMC1154** | *h^-^ ade6-M26 rec14-cdk2-GFP::KanMX6* | Fig S9 |
| **CMC1155** | *h^+^ ade6-3049 rec14-cdk2-GFP::KanMX6* | Fig S9 |
| **CMC1158** | *h^-^ ade6-M26 rec14-cdk1 cdk2-GFP::KanMX6* | Fig S9 |
| **CMC1159** | *h^+^ ade6-3049 rec14-cdk1 cdk2-GFP::KanMX6* | Fig S9 |
| **CMC1165** | *h^-^ pat1-114 leu1-32 ura4^-^ rad3::ura4^+^ cdc2-asM17* | Fig S5 |
| **CMC1177** | *h^-^/h^-^ pat1-114/pat1-114 ade6-M210/ade6-M216 leu1-32/leu1-32 crs1::hphMX6/crs1::hphMX6 rad50S/rad50S* | Fig S3 |
| **CMC1192** | *h^-^/h^-^ pat1-114/pat1-114 leu1-32/leu1-32 ade6-M210/ade6-M216 cdc2-asM17/cdc2-asM17 rec25-GFP::KanMX6/rec25-GFP::KanMX6* | Fig 7, 8, and 9 |
| **CMC1207** | *h^-^/h^-^ pat1-114/pat1-114 leu1-32/leu1-32 ade6-M210/ade6-M216 crs1::hphMX6/crs1::hphMX6 cig1::hphMX6/cig1::hphMX6 rec25-GFP::KanMX6/rec25-GFP::KanMX6* | Fig 6, 7, and S6 |
| **CMC1210** | *h^-^ ade6-M26 rec7-cdk1cdk2* | Fig S10 |
| **CMC1211** | *h^+^ ade6-3049 rec7-cdk1cdk2* | Fig S10 |
| **CMC1216** | *h^-^ ade6-M26 rec27-cdk* | Fig S10 |
| **CMC1219** | *h^+^ ade6-3049 rec14-cdk1cdk2* | Fig S10 |
| **CMC1220** | *h^-^ ade6-M26 rec14-cdk1cdk2* | Fig S10 |
| **CMC1225** | *h^-^ ade6-M26 rec7-cdk1cdk2 rec14-cdk1cdk2* | Fig S10 |
| **CMC1226** | *h^-^ ade6-M26 rec7-cdk1cdk2 rec27-cdk* | Fig S10 |
| **CMC1230** | *h^+^ ade6-3049 rec7-cdk1cdk2 rec14-cdk1cdk2* | Fig S10 |
| **CMC1231** | *h^-^ ade6-M26 rec14-cdk1cdk2 rec27-cdk* | Fig S10 |
| **CMC1242** | *h^-^ ade6-M26 rec7-cdk1cdk2 rec14-cdk1cdk2 rec27-cdk* | Fig S10 |
| **CMC1244** | *h^+^ ade6-3049 rec27-cdk* | Fig S10 |
| **CMC1261** | *h^-^ ade6-M26 rec14-cdk total* | Fig S9 |
| **CMC1262** | *h^+^ ade6-3049 rec14-cdk total* | Fig S9 |
| **CMC1264** | *h^+^ ade6-3049 rec7-cdk1cdk2 rec27-cdk* | Fig S10 |
| **CMC1265** | *h^+^ ade6-3049 rec14-cdk1cdk2 rec27-cdk* | Fig S10 |
| **CMC1266** | *h^-^ ade6-M26 rec10-cdk total* | Fig S9 |
| **CMC1267** | *h^+^ ade6-3049 rec10-cdk total* | Fig S9 |
| **CMC1270** | *h^+^ ade6-3049 rec7-cdk1cdk2 rec14-cdk1cdk2 rec27-cdk* | Fig S10 |
| **CMC1322** | *h^-^ leu1-32::pJK148 (empty) ade6-M26* | Fig 10 |
| **CMC1324** | *h^+^ leu1-32::pJK148 (empty) ade6-3049* | Fig 10 |
| **CMC1325** | *h^-^ crs1::hphMX6 leu1-32::pJK148 (empty) ade6-M26* | Fig 10 |
| **CMC1326** | *h^+^ crs1::hphMX6 leu1-32::pJK148 (empty) ade6-3049* | Fig 10 |
| **CMC1328** | *h^-^ crs1::hphMX6 leu1-32::pJK148 cdc13 ade6-M26* | Fig 10 |
| **CMC1330** | *h^+^ crs1::hphMX6 leu1-32::pJK148 cdc13 ade6-3049* | Fig 10 |
| **CMC1332** | *h^-^ crs1::hphMX6 leu1-32::pJK148 cdc2 ade6-M26* | Fig 10 |
| **CMC1334** | *h^+^ crs1::hphMX6 leu1-32::pJK148 cdc2 ade6-3049* | Fig 10 |
| **CMC1336** | *h^-^ crs1::hphMX6 leu1-32::pJK148 crs1 ade6-M26* | Fig 10 |
| **CMC1338** | *h^+^ crs1::hphMX6 leu1-32::pJK148 crs1 ade6-3049* | Fig 10 |
| **CMC1345** | *h^-^ leu1-32::pJK148 (empty) ade6-M26 (two copies)* | Fig 10 |
| **CMC1347** | *h^+^ leu1-32::pJK148 (empty) ade6-3049 (two copies)* | Fig 10 |
| **CMC1348** | *h^-^ crs1::hphMX6 leu1-32::pJK148 (empty) ade6-M26 (two copies)* | Fig 10 |
| **CMC1349** | *h^+^ crs1::hphMX6 leu1-32::pJK148 (empty) ade6-3049 (two copies)* | Fig 10 |
| **CMC1351** | *h^-^ crs1::hphMX6 leu1-32::pJK148 cdc13 ade6-M26 (two copies)* | Fig 10 |
| **CMC1353** | *h^+^ crs1::hphMX6 leu1-32::pJK148 cdc13 ade6-3049 (two copies)* | Fig 10 |
| **CMC1359** | *h^-^ crs1::hphMX6 leu1-32::pJK148 crs1 ade6-M26 (two copies)* | Fig 10 |
| **CMC1360** | *h^+^ crs1::hphMX6 leu1-32::pJK148 crs1 ade6-3049 (two copies)* | Fig 10 |
| **CMC1366** | *h^-^ crs1::hphMX6 leu1-32::pJK148 puc1 ade6-M26* | Fig 10 |
| **CMC1368** | *h^+^ crs1::hphMX6 leu1-32::pJK148 puc1 ade6-3049* | Fig 10 |
