# Supplementary material for: CDK contribution to DSB formation and recombination in fission yeast meiosis
Source: PLoS Genet. 2019 Jan 14;15(1):e1007876. doi: 10.1371/journal.pgen.1007876 (PMC6331086; doi:10.1371/journal.pgen.1007876)
Supplement: S2 Table — (DOCX) [file pgen.1007876.s012.docx]

| **Name** | **Sequence (5’-3’)** | | **Used for** |
| --- | --- | --- | --- |
| **cig1-D1** | **tacctgagtaaaaggcagtttgaaaaccgactttcatcgcatgtccaaaactttctaaacaagtgtgtgctggtttacta**cGTACGCTGCAGGTCGAC | | *cig1::hphMX6* deletion construction |
| **cig1-D2** | **atccgtaataataaatatcgtaggcttaataatagcaaactaactcagaatatcattgttaacaacttctgaaagcaaac**ATCGATGAATTCGAGCTCG | | *cig1::hphMX6* deletion construction |
| **crs1-D1** | **CTTAGAAAATCACTATTGAATTTCTTAATTCAAACAAACATCCTTATTCTAATAATCATTTAAATTATAAAATTTATGAA**cGTACGCTGCAGGTCGAC | | *crs1::hphMX6* deletion construction |
| **crs1-D2** | **GGGTTTAGAGTTTTGATTAAATAGCTTAGTTTTAATAATTAAGTTCATTAGAATTTGGGTTAATAGATGGGTTTGAAGCT**ATCGATGAATTCGAGCTCG | | *crs1::hphMX6* deletion construction |
| **rec7-D1** | **caccgtgacggaacgtactgcgttatcagataaatgaggttcagtctaacgcgtcttctcatattcaaacataaacaaac**CGTACGCTGCAGGTCGAC | | *rec7::ura4^+^* deletion construction |
| **rec7-D2** | **aaataaccattccgatcctgatttcgttccatttttactacttttttaaatttgggattaagttgaattggtctgtcttc**ATCGATGAATTCGAGCTCG | | *rec7::ura4^+^* deletion construction |
| **rec10-D1** | **ATCAACTGAAACCGTTTTACGTATTTAATCCTATTTATTATTCCAAAAAAAATCTATTAACACTTAAACGTAACTAAAAT**CGTACGCTGCAGGTCGAC | | *rec10::ura4^+^* deletion construction |
| **rec10-D2** | **TAACGCTTCCAATGTGAATTGTTTTAAAGTAATATGTCAATATAAGCCAATATCAATAAATTTGATACTATAGGTTCAAG**ATCGATGAATTCGAGCTCG | | *rec10::ura4^+^* deletion construction |
| **rec14-D1** | **taagtattataaagtattggaaagcgccagcattttaattactaattttgttgtattattttccttatttattagatact**cGTACGCTGCAGGTCGAC | | *rec14::ura4^+^* and *rec14::hphMX6* deletion constructions |
| **rec14-D2** | **CCAAATATTAGAAACTCCTTAATTTTTTGCTATTTTTTAAAAACTTTTCCATCGGTCATTAAGAAAGCACAAGAGTGCAA**ATCGATGAATTCGAGCTCG | | *rec14::ura4^+^* and *rec14::hphMX6* deletion constructions  cassette amplification for transformation to generate *rec14* mutants (G-418 resistant GFP-versions) |
| **rec27-D1** | **AATTTATCACATTAACTATTGAATAATGAAACGCGTTTACAGGTATCGCATTGTCAAGTATCTGTTGTATATTTTAGCAT**CGGATCCCCGGGTTAATTAA | | *rec27::ura4^+^* deletion construction |
| **rec27-D2** | **TAAATTGTATGCTCATAACATATTTTTAATTCGTTTTATGATTTTATGGCACTAGTTTATATAATGTGTTTAAAATGACT**GAATTCGAGCTCGTTTAAAC | | *rec27::ura4^+^* deletion construction  cassette amplification for transformation to generate *rec27* mutants (Hygromycin B resistant GFP-versions) |
| For the oligos on top **bold** indicates *S. pombe* genome sequence | | | |
| **rec7-cdk1F** | | GGTGATAGCTGCAGCACCTTTTAATCCC | *rec7-cdk1* site-directed mutagenesis |
| **rec7-cdk1R** | | GGGATTAAAAGGTGCTGCAGCTATCACC | *rec7-cdk1* site-directed mutagenesis |
| **rec7-cdk2F** | | GCTGCTGCGCCGCTGAACTCTCAG | *rec7-cdk2* site-directed mutagenesis |
| **rec7-cdk2R** | | CTGAGAGTTCAGCGGCGCAGCAGC | *rec7-cdk2* site-directed mutagenesis |
| **rec14-cdk1F** | | GAAATAGATGCCCCTCATAAACTTGGCGTTC | *rec14-cdk1* site-directed mutagenesis |
| **rec14-cdk1R** | | GAACGCCAAGTTTATGAGGGGCATCTATTTC | *rec14-cdk1* site-directed mutagenesis |
| **rec14-cdk2F** | | GCCACACTGCCCCTGTACGATCAGTGG | *rec14-cdk2* site-directed mutagenesis |
| **rec14-cdk2R** | | CCACTGATCGTACAGGGGCAGTGTGGC | *rec14-cdk2* site-directed mutagenesis |
| **rec27-cdkF** | | GTTGTTAAAGCCCCAATGAACCAACCAAC | *rec27-cdk* site-directed mutagenesis |
| **rec27-cdkR** | | GTTGGTTGGTTCATTGGGGCTTTAACAAC | *rec27-cdk* site-directed mutagenesis |
| **rec7-3** | | AAAGTAATACACCGTGACGG | cassette amplification for transformation to generate *rec7* mutants |
| **rec7-STOP** | | AAATAACCATTCCGATCCTGATTTCGTTCCATTTTTACTACTTTTTTAAATTTGGGATTAAGTTGAATTGGTCTGTCTTCCTACATCTTGTTCCATACCC | cassette amplification for transformation to generate *rec7* mutants (unmarked and untagged versions) |
| **rec14-1** | | TATCTAAGGAGAACGCAACC | cassette amplification for transformation to generate *rec14* mutants |
| **rec14-STOP** | | CCAAATATTAGAAACTCCTTAATTTTTTGCTATTTTTTAAAAACTTTTCCATCGGTCATTAAGAAAGCACAAGAGTGCAATTATTCTGTAGCAGCAGCTC-3’ | cassette amplification for transformation to generate *rec14* mutants (unmarked and untagged versions) |
| **rec27-1** | | CCGTTTAAGTACCTTGCAGC | cassette amplification for transformation to generate *rec27* mutants |
| **rec27-STOP** | | TAAATTGTATGCTCATAACATATTTTTAATTCGTTTTATGATTTTATGGCACTAGTTTATATAATGTGTTTAAAATGACTTTATACTTCTGTATTTGTTGGTTGG | cassette amplification for transformation to generate *rec27* mutants (unmarked and untagged versions) |
| **mbs1-15** | | CATCACCCCTTATCTTAGGC | Probe for Southern blot |
| **mbs1-16** | | TAGTATTCAGGTGCGCGAGG | Probe for Southern blot |
| **puc1-SacI** | | CCGATGAGCTCATACATTCG | *puc1* cloning |
| **puc1-KpnI** | | ATGCGGTACCCAACTATCGTGC | *puc1* cloning |
| **crs1-SalI(3)** | | ACTGGTCGACGCACAGAAGACC | *crs1* cloning |
| **crs1-EcoRI(3)** | | GTCAGAATTCGTATGATAGCGAG | *crs1* cloning |
| **pJK148-1** | | cacagcttgtctgtaagcgg | pJK148 integrants analysis |
| **pJK148-2** | | GCGGTATTTTCTCCTTACGC | pJK148 integrants analysis |
| **pJK148-up KpnI** | | CCCGATTTAGAGCTTGACG | pJK148 integrants analysis |
| **pJK148-down BamHI** | | CACAGGAAACAGCTATGACC | pJK148 integrants analysis |
| **cdc13-1** | | TTGAATCATAGGGAAGGAGC | pJK148 integrants analysis |
| **cdc2-10** | | CCTTTGTACGAATCAAGAGC | pJK148 integrants analysis |
| **puc1-2** | | CTTCTATGGAATCCTATCGC | pJK148 integrants analysis |
| **crs1-3** | | GCTTCACGAGTATTGGGAGC | pJK148 integrants analysis |
| **leu1-3** | | GAGGCTTCTGTCATTCCTGG | qPCR (pJK148 copy number) |
| **leu1-4** | | TACCGTGAATGGGCTCAACC | qPCR (pJK148 copy number) |
| **mde2-3** | | GAAAGCCAAACATCCTCTGG | qPCR (pJK148 copy number) |
| **mde2-4** | | AACGTTCGAATGGTTGGACT | qPCR (pJK148 copy number) |
